# Suppression of Phytochrome-Interacting Factors Enhances Photoresponses of Seedlings and Delays Flowering With Increased Plant Height in *Brachypodium distachyon*

**DOI:** 10.3389/fpls.2021.756795

**Published:** 2021-09-28

**Authors:** Quyen T. N. Hoang, Sharanya Tripathi, Jae-Yong Cho, Da-Min Choi, Ah-Young Shin, Suk-Yoon Kwon, Yun-Jeong Han, Jeong-Il Kim

**Affiliations:** ^1^Department of Integrative Food, Bioscience and Biotechnology, Chonnam National University, Gwangju, South Korea; ^2^Plant Systems Engineering Research Center, Korea Research Institute of Bioscience and Biotechnology, Daejeon, South Korea; ^3^Kumho Life Science Laboratory, Chonnam National University, Gwangju, South Korea

**Keywords:** *Brachypodium distachyon*, phytochrome-interacting factors, DNA-binding, sequestration, flowering, chlorophyll biosynthesis

## Abstract

Phytochromes are red and far-red photoreceptors that regulate plant growth and development under ambient light conditions. During phytochrome-mediated photomorphogenesis, phytochrome-interacting factors (PIFs) are the most important signaling partners that regulate the expression of light-responsive genes. However, the function of PIFs in monocots has not been studied well. In this study, using RNA interference (RNAi), we investigated the functions of *BdPIL1* and *BdPIL3*, two PIF-like genes identified in *Brachypodium distachyon*, which are closely related to Arabidopsis *PIF1* and *PIF3*. The expression of their genes is light-inducible, and both BdPIL1 and BdPIL3 proteins interact with phytochromes in an active form-specific manner. Transgenic Brachypodium seedlings with the RNAi constructs of *BdPIL1* and *BdPIL3* showed decreased coleoptile lengths and increased leaf growth when exposed to both red and far-red light. In addition, the transgenic plants were taller with elongated internodes than wild-type Bd21-3 plant, exhibiting late flowering. Moreover, RNA-seq analysis revealed downregulation of many genes in the transgenic plants, especially those related to the regulation of cell number, floral induction, and chlorophyll biosynthesis, which were consistent with the phenotypes of increased plant height, delayed flowering, and pale green leaves. Furthermore, we demonstrated the DNA-binding ability of BdPIL1 and BdPIL3 to the putative target promoters and that the DNA-binding was inhibited in the presence of phytochromes. Therefore, this study determines a molecular mechanism underlying phytochrome-mediated PIF regulation in Brachypodium, i.e., sequestration, and also elucidates the functions of BdPIL1 and BdPIL3 in the growth and development of the monocot plant.

## Introduction

Light is an important signal for plant photomorphogenesis, which is mediated by various photoreceptors, including phytochromes (Legris et al., 2019). Phytochromes are red (R) and far-red (FR) light-absorbing photoreceptors that regulate plant growth and development in response to ambient light conditions (Tripathi et al., 2019). They are biosynthesized in the inactive R light-absorbing form (Pr), which is converted into the physiologically active FR light-absorbing form (Pfr) upon exposure to red light. The Pr-to-Pfr photoactivation induces highly regulated signaling processes in plants, resulting in transcriptional reprogramming in response to the environmental signals (Cheng et al., 2021). In phytochrome-mediated light signaling pathways, various transcription factors play roles in bridging the photoactivation of phytochromes with the expression of light-responsive genes (Jing and Lin, 2020).

PHYTOCHROME INTERACTING FACTORs (PIFs), which belong to the basic helix-loop-helix (bHLH) family of transcription factors containing conserved active phytochrome-binding (APB) motifs in the N-terminal domain, play key roles in the phytochrome-mediated photomorphogenesis (Leivar and Quail, 2011; Paik et al., 2017). Thus far, eight PIFs have been identified in *Arabidopsis thaliana* (Pham et al., 2018; Oh et al., 2020). Phytochromes interact with PIFs in a Pfr-specific manner, inhibit the binding of PIFs to the target promoters (i.e., sequestration), and induce protein degradation of PIFs (Al-Sady et al., 2006; Park et al., 2012, 2018; Shin et al., 2016), thereby mediating various photomorphogenic developmental processes, such as seed germination, seedling de-etiolation, chlorophyll biosynthesis, and flowering. For example, phytochromes stimulate seed germination via the negative regulation of PIF1 that functions in inhibiting seed germination (Oh et al., 2004). PIF3 promotes and maintains skotomorphogenic development (i.e., etiolated seedlings in the dark) by repressing photomorphogenesis; thus, phytochromes induce seedling de-etiolation by inhibiting the functions of PIF3 (Kim et al., 2003; Pham et al., 2018). Recently, phytochromes have been shown to respond not only to light but also to other important environmental cues, such as temperature, by regulating PIF4 (Jung et al., 2016; Legris et al., 2016). Therefore, phytochrome-PIF signaling modules play important roles in the regulation of plant photomorphogenesis (Hoang et al., 2019; Favero, 2020).

*Brachypodium distachyon* (hereafter, Brachypodium) has been developed as a model system for temperate grasses, cereals, and bioenergy crops (Draper et al., 2001; Opanowicz et al., 2008). With the completion of genome sequencing (The International Brachypodium Initiative, 2010), Brachypodium is now widely used as a monocot model plant (Girin et al., 2014; Scholthof et al., 2018). As many crops and cereals are monocots, it is necessary to study the functions of PIFs in monocot plants to improve their productivity. Especially, the functional importance of PIFs is increasing with unraveling new functional roles of PIFs in signal integration from multiple processes (Paik et al., 2017). However, most of the investigations on PIFs have been performed in Arabidopsis. In contrast, PIF functions in monocot plants have not been studied well. In this regard, we intended to investigate the functional roles of PIFs in Brachypodium.

Here, we characterized two *B. distachyon* PIF-like (BdPIL) proteins, which are homologous to Arabidopsis PIF1 and PIF3 (hereafter, BdPIL1 and BdPIL3, respectively). Initially, we verified the interaction of BdPIL1 and BdPIL3 proteins with phytochromes in a Pfr-specific manner. Then, we investigated the physiological functions of BdPIL1 and BdPIL3 using transgenic Brachypodium plants with RNA interference (RNAi) constructs, which included responses to R and FR light and their apparent growing phenotypes. To account for the observed physiological functions of BdPIL1 and BdPIL3, RNA-seq analysis was performed to determine the differentially expressed genes (DEGs) between wild-type (i.e., inbred line Bd21-3) and the RNAi-suppression plants. Moreover, we confirmed the DNA-binding ability of BdPIL1 and BdPIL3 to the promoters of putative target genes, and more importantly, we verified that the DNA-binding ability was inhibited in the presence of phytochromes. Therefore, the present study provides a molecular mechanism for the regulation of monocot PIFs by phytochromes, in addition to the functional roles of PIFs in Brachypodium.

## Materials and Methods

### Yeast Two-Hybrid Assay

Full length cDNA of *Bradi1g13980* (BdPIL1; 445 aa) and *Bradi2g11100* (BdPIL3; 549 aa) was cloned into pGADT7 vector and fused with the GAL4 activation domain at the C-terminus, and either the N-terminal domain (BdAN, 1∼612 aa; BdBN, 1∼660 aa) or C-terminal domain (BdAC, 568∼1131 aa; BdBC, 653∼1181 aa) of BdphyA (*Bradi1g10520*) or BdphyB (*Bradi1g64360*) were cloned into the pGBKT7 vector and fused with the GAL4 DNA-binding domain at the C-terminus. The primers used for cloning are listed in Supplementary Table 1. The vector constructs were then transformed into the yeast strain Y2H Gold (for pGBKT7 constructs) or Y187 (for pGADT7 constructs) using the lithium acetate transformation method (Gietz and Schiestl, 2007) and plated on synthetic dextrose without tryptophan (SD-Trp) or leucine (SD-Leu), respectively. After mating, the yeast cells were selected on SD-Trp-Leu (DDO) plates, and the selected cells were plated on SD-His-Trp-Leu-Ade/X-gal/Aureobasidin A (QDO/X/A) to analyze protein–protein interactions. For quantitative analysis, β-galactosidase assay was performed using the mated yeast cells, according to the Yeast Protocol Handbook (Clontech).

To determine whether the interaction was Pfr-specific, full-length cDNA of BdphyA and BdphyB were cloned into the pGBKT7 vector and used for Y2H assays in the presence of phycocyanobilin (PCB) as chromophore. The mated yeast cells were plated on non-selective (DDO) or selective (QDO/X/A) media containing 20 μM PCB, and incubated for 3 days in the dark or under continuous R light (3 μmol⋅m^–2^⋅s^–1^), representing the Pr and Pfr forms, respectively.

### Preparation of Recombinant Proteins

Full-length recombinant BdphyA, oat phytochrome A (AsphyA), and *Arabidopsis thaliana* phyB (AtphyB), with a 10-amino acid streptavidin affinity-tag (strep-tag; SAWRHPQFGG) at the C-terminus, were prepared using the *Pichia pastoris* protein expression system, as previously reported (Shin et al., 2016; Han et al., 2019). PCB was added (final concentration: 20 μM) before purification using streptavidin affinity chromatography under dim green light, and zinc fluorescence assay was performed to verify the ligation of PCB to the phytochromes. The purified phytochrome (as the Pr form) was exposed to R light to generate the Pfr form, which was verified using a diode array UV-Visible spectrophotometer (Cary; Varian Inc).

To prepare recombinant BdPIL1 and BdPIL3, full-length cDNA was cloned into the pGEX 4T-1 (GE Healthcare) vector with the strep-tag, named pStrep vector (Choi et al., 2021), using the primers listed in Supplementary Table 1. Glutathione S-transferase and streptavidin (GST/strep) tags were fused to the N- and C-termini of the recombinant proteins, respectively. The *E. coli* strain BL21-CodonPlus^TM^ (Agilent Technology) was used for protein expression and the GST/strep-tagged recombinant BdPIL1 and BdPIL3 proteins were purified using streptavidin affinity chromatography.

### *In vitro* Protein–Protein Interaction Assay

Pull-down experiments were performed to examine the *in vitro* protein–protein interaction between phytochromes and BdPILs, as previously described (Jeong et al., 2016; Shin et al., 2016). To 1 mL of pull-down buffer (100 mM Tris–HCl, pH 7.8, 1 mM EDTA, 150 mM NaCl, and 100 μg mL^–1^ BSA), 2 μg of phytochrome (Pr or Pfr form) and 2 μg of GST/strep-tagged BdPIL1 or BdPIL3 were mixed and incubated for 60 min at 4°C with gentle rotation. Next, 50 μL of glutathione resin was added, and the solution was incubated for 30 min. After washing and pelleting glutathione bead-bound proteins, AsphyA/BdphyA, AtphyB, and GST/strep-fused BdPIL1/BdPIL3 proteins were detected using 1:5,000 AsphyA-specific monoclonal antibody (oat25), 1:2,000 AtphyB polyclonal antibody (aN-20; Santa Cruz Biotechnology), and 1:2,000 GST-specific monoclonal antibody (sc-138; Santa Cruz Biotechnology), respectively.

### Expression Analysis of *BdPIL1* and *BdPIL3*

To determine whether the expression of *BdPIL1* and *BdPIL3* was light-inducible, the lemma of Brachypodium inbred line Bd21-3 (wild-type) seeds were removed and surface-sterilized using 70% (v/v) ethanol and 2% (w/v) sodium hypochlorite for 5 min each with gentle shaking. After washing with sterile distilled water, the seeds were placed on half-strength Murashige and Skoog (MS) medium plates containing 0.8% phytoagar (pH 5.8). After 7 days of cold and dark treatment, the seeds were exposed to white light (WL) for 12 h at 22°C to induce germination. Five days after growth in the dark (D), the seedlings were exposed to WL (100 μmol⋅m^–2^⋅s^–1^) for 1 or 4 h before harvesting. Moreover, to investigate light-inducible expression under different light conditions, the dark-grown seedlings were exposed to R (λ_*max*_ = 654 nm; bandwidth = 25 nm; intensity = 20 μmol⋅m^–2^⋅s^–1^), FR (λ_*max*_ = 738 nm and bandwidth = 42 nm; intensity = 20 μmol⋅m^–2^⋅s^–1^), and blue (B; λ_*max*_ = 450 nm and bandwidth = 20 nm; intensity = 20 μmol⋅m^–2^⋅s^–1^) light for 4 h in an LED growth chamber (Vision Science Co., Korea). To investigate tissue-specific expression, Bd21-3 plants were grown under long day conditions (18-h light/6-h dark cycle) at 22°C. Root, stem, and leaf tissues were collected from 4-week-old plants, and flower tissues were collected from 6-week-old plants.

For gene expression analysis, seedling and tissue samples were frozen in liquid nitrogen immediately after collection, total RNA was isolated using RNAiso Plus (Takara Bio), and cDNA was synthesized using RNA to cDNA EcoDry Premix kit (Takara Bio). To determine the expression levels of *BdPIL1* and *BdPIL3*, qRT-PCR was performed using Stratagene Mx3005P with Brilliant III Ultra-Fast SYBR Green Q-PCR Master Mix (Agilent Technologies) and corresponding primers (Supplementary Table 1). The expression of *BdUBC18*, a housekeeping gene, was used for data normalization, and the relative expression levels were estimated by setting the transcript level in the dark-grown or root samples as 1.

### Generation of Transgenic Brachypodium Plants

To determine the role of BdPIL1 and BdPIL3, we investigated the phenotype of transgenic Bd21-3 plants transformed with RNAi constructs. For this, we cloned partial sequences of *BdPIL1* (68∼550 bp) and *BdPIL3* (152∼685 bp), based on a previous report (Kerschen et al., 2004), into the pFGC5941 vector using the corresponding primers listed in Supplementary Table 1. The interference fragments were cloned into the RNAi vector in the opposite orientation, separated by the *ChsA* intron. The binary vector constructs were then transformed into *Agrobacterium tumefaciens* strain AGL1, and Brachypodium transformation was performed using embryogenic calli induced from immature embryos, according to a previously described method (Alves et al., 2009). The transformed plantlets with well-developed roots were then transferred to the soil, grown under long day conditions for 2 weeks, and sprayed with 0.8% (v/v) BASTA^®^ to select putative transgenic plants. Herbicide resistance was determined after 7 days, and herbicide-resistant plants were further analyzed using PCR with total genomic DNA isolated from the leaves of mature plants. The coding regions of the 35S promoter (P_35__*S*_)-*BdPIL1* or P_35__*S*_-*BdPIL3* and *BAR* were PCR-amplified using the primers listed in Supplementary Table 1. *BdUBC18* was also PCR-amplified using the same template to serve as loading controls.

To obtain homozygous lines with a single transgene integration, the herbicide-resistant plants exhibiting 3:1 segregation in the T2 generation were selected (Supplementary Tables 2, 3), and the plants from the T3 or T4 generations were used for subsequent analyses. To verify RNAi-suppression of *BdPIL1* and *BdPIL3* in the homozygous lines, 5-day-old dark-grown seedlings were transferred into the growth chamber, exposed to WL for 4 h, and harvested for qRT-PCR analysis. *BdUBC18* expression levels were used for data normalization, and relative expression levels were estimated by setting the transcript level in Bd21-3 as 1.

### Photoresponse and Phenotypic Analyses

For the photoresponse analysis, seeds without lemma were surface-sterilized, stratified at 4°C for 7 days in the dark, and the embryos were placed vertically on 0.8% phytoagar plates containing half-strength MS salts and vitamins. The seedlings were then exposed to WL for 12 h to promote germination, returned to darkness at 22°C for 1 day, and grown further for 5 days in the dark (D), under continuous R light (cR, 20 μmol⋅m^–2^⋅s^–1^) or continuous FR light (cFR, 20 μmol⋅m^–2^⋅s^–1^). Subsequently, the lengths of the coleoptile and first and second leaves of the seedlings were measured using ImageJ.

For the phenotypic analysis, Brachypodium plants were grown at 22°C in a culture room under long day conditions (18-h light/6-h dark cycle), and flowering time, plant height, and chlorophyll content were measured. The flowering time was estimated from germination to the day when the spike emerged (i.e., days to heading), and plant height was measured from the base to the highest point of the plant. Total chlorophyll content was determined using the second and third leaves of the primary tillers of 8-week-old plants. 100 mg of leaf sample was ground in liquid nitrogen, and incubated in 1 mL of 80% acetone, with overnight-shaking in the dark. Absorbances were measured using the UV-Visible spectrophotometer (Cary), and the total chlorophyll content was estimated using the equation: chlorophyll_*a+b*_ = 7.15 × A_660_ + 18.71 × A_647_. In addition, stem internode numbers and lengths were also measured. The internode number was counted using the highest tiller, in which the internode lengths were measured after removing the leaf sheath.

### Histochemical Staining

Considering the increased height exhibited in the *BdPIL1*/RNAi and *BdPIL3*/RNAi plants, histochemical analysis was performed to determine the number and size of cells using the cross-sections of the first internode of the highest tiller. The edge of the first internode was excised using scalpel blade No. 11 (Sigma-Aldrich), subjected to 0.02% toluidine blue staining for 30 s, and rinsed with distilled water. Stem diameter, the number of vascular bundles and pitch cells, and pitch cavity were then estimated, as previously described (Matos et al., 2013; Sakai et al., 2021).

### RNA-Seq Analysis

Bd21-3 and transgenic plants harboring the RNAi constructs of *BdPIL1* and *BdPIL3* (*BdPIL1*/RNAi and *BdPIL3*/RNAi, respectively) were grown for 4 weeks under long day conditions, and leaf samples were collected for RNA-seq analysis at 4 h after the start of light cycle. RNA concentration and purity were determined using NanoDrop 2000 (Thermo Fisher Scientific), and TruSeq RNA Sample Preparation Kit V2 (Illumina) was used for library construction. Sequencing was performed using a HiSeq4000 platform (Illumina) with 150-nt paired end sequencing at Macrogen (South Korea). FastQC v.0.11.4 was used for the quality examination of the paired-end reads. Cutadapt v.1.15 and Sickle v.1.33 were used to filter low-quality reads and adaptors. After trimming, reads were aligned to the Phytozome 9.0 *B. distachyon* reference genome (Bdistachyon_192_hardmasked.fa.gz) using TopHat2 version 2.0.10 with default parameters. Cufflinks version 2.2.1 was used to calculate FPKM (Fragments Per Kilobase of transcripts per Million mapped reads) values. The cuffdiff was carried out for the selection of DEGs (fold change ≥ 2). Gene Ontology and KEGG pathway enrichment analyses were performed using DAVID ver. 6.8 and CluGO ver. 2.5.5 in cytoscape ver. 3.7.1. Heat maps, Venn diagram, and hierarchical clustering were performed with R scripts.

To validate RNA-seq results, we selected several genes involved in the regulation of elongated growth and cell number, including *Bradi1g28120* (LOC100838644), *Bradi1g51490* (LOC100828255), *Bradi2g24980* (LOC100830094), *Bradi2g57027* (LOC100823873), *Bradi3g46930* (LOC100840545), and *Bradi4g10290* (LOC104584443), and performed qRT-PCR using RNA extracted from 4-week-old or 8-week-old plant leaves and the corresponding primers listed in Supplementary Table 1.

### Gene Expression Analysis Related to Flowering and Chlorophyll Biosynthesis

To account for the delayed flowering in *BdPIL1*/RNAi and *BdPIL3*/RNAi plants, we analyzed the expression of Brachypodium genes that induce flowering, *FLOWERING LOCUS T* (*BdFT1*) and *CONSTANS* (*BdCO1*) (Lv et al., 2014; Feng et al., 2017; Qin et al., 2019). As the expression of these genes is regulated by diurnal rhythm, we cultivated Brachypodium plants under long day conditions for 8 weeks, and leaf samples were harvested every 2 h in a day. After RNA extraction and RT-PCR, the expression levels of these genes were quantified from DNA gel images with normalization using *BdUBC18* expression levels. Then, qRT-PCR was used to analyze the transcript levels in the samples harvested at ZT8 for *BdFT1* and ZT20 for *BdCO1*. The primers used for these analyses are listed in Supplementary Table 1.

To compare chlorophyll biosynthesis between Bd21-3 and the RNAi plants, we analyzed the expression levels of two important genes in chlorophyll biosynthesis, *GLU-tRNA REDUCTASE* (*BdHEMA1*; Bradi3g30160) and *PROTOCHLOROPHYLLIDE OXIDOREDUCTASE* (*BdPOR*; Bradi5g26230), which were identified using BLASTP with corresponding Arabidopsis proteins as the query sequences. qRT-PCR was performed using RNA samples extracted from 5-day-old dark-grown seedlings and the corresponding primers for *BdHEMA1* and *BdPOR* (Supplementary Table 1).

### Electrophoretic Mobility Shift Assay

To determine the DNA-binding ability of BdPIL1 and BdPIL3, EMSA was performed as previously reported (Moon et al., 2008). The sequences of 3 kb upstream region of three genes (Bradi1g51490/*BdSAUR50*, Bradi5g26230/*BdPOR*, and *BdMIR156H*/LOC104794734) were analyzed using PlantPAN 3.0, and 60-bp promoter fragments containing a G-box (CACGTG), E-box (CANNTG), or N-box (CACG(A/C)G) sequence that was scored over 0.98 for the binding to BdPIL1 and BdPIL3 were selected as probes (Supplementary Table 1). After labeling the probes with ^32^P-ATP using DNA 5′-End-Labeling System (Promega), 1 pmol of the ^32^P-labeled probe was incubated with 2 μg of BdPIL1 or BdPIL3 in a 20 μL reaction mixture (50 mM Tris–HCl, pH 7.5, 50 mM NaCl, 200 mM KCl, 5 mM MgCl_2_, 5 mM EDTA, 5 mM DTT, and 250 mM BSA) for 30 min. Cold competitor probes were generated from dimerized oligos without labeling. The reaction mixtures were resolved on 5% native polyacrylamide gels and dried under vacuum before autoradiography.

To examine the effect of phytochrome interaction on the DNA-binding ability of BdPIL1 or BdPIL3, different amounts (0.1, 0.5, 1, 1.5, and 2 μg) of BdphyA or AtphyB (in the Pfr form) were added to the reaction mixture containing 2 μg of BdPIL1 or BdPIL3 before EMSA. For negative control, 2 μg of the phytochrome protein was incubated with 1 pmol of the ^32^P-labeled probe only. In addition, to quantify the DNA-binding ability of BdPIL1 and BdPIL3 in the presence of phytochromes, the DNA probe was stained using Electrophoretic Mobility-Shift Assay Kit with SYBR^TM^ Green and SYPRO^TM^ Ruby EMSA stains (Thermo Fisher Scientific). The relative DNA binding was estimated by setting the signal from BdPIL1-DNA or BdPIL3-DNA complex in the sample without phytochromes as 1.

### Statistical Analysis

Analysis of variance and Duncan’s multiple range test were performed to determine the significant differences in multiple comparisons using IBM SPSS Statistics 20 software. The significant differences in mean values were compared using LSD at *P* < 0.05 (labeled ‘^∗^’) or *P* < 0.01 (labeled ‘^∗∗^’).

### Accession Numbers

Accession numbers to the referenced genes are in Supplementary Table 4. The RNA-seq data included in this study were deposited into Korean Bioinformation Center (KOBIC)^1^ under the accession codes of KBRS20191011_0000024 to KBRS20191011_0000032.

## Results

### Phytochrome-Interacting Factors in Brachypodium

Among 146 bHLH transcriptional factors in the genome of Brachypodium (Niu et al., 2017), we identified the following five *B. distachyon* PIF-like (*BdPIL*) genes containing conserved active phytochrome binding (APB) and bHLH motifs using BLASTP analysis with Arabidopsis PIFs as query sequences (Supplementary Figures 1A,B): Bradi1g13980, Bradi2g11100, Bradi1g58230, Bradi1g06670, and Bradi5g33170. They belong to the sixth subfamily of Brachypodium bHLH transcriptional factors in the 24 phylogenetic groups classified previously (Niu et al., 2017). As PIF1 and PIF3 are the most studied Arabidopsis PIFs (Pham et al., 2018), we selected two Brachypodium PIFs that are closely related to PIF1 and PIF3 from the phylogenetic analysis (Supplementary Figure 1C), i.e., Bradi1g13980 (BdPIL1) and Bradi2g11100 (BdPIL3) for the present study.

**FIGURE 1:**
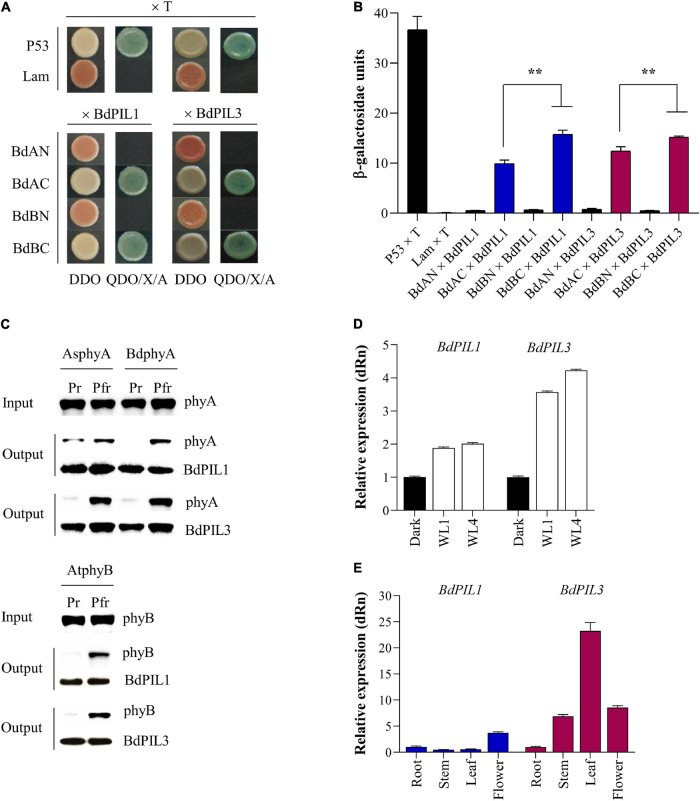
Isolation and expression analysis of two *Brachypodium distachyon* phytochrome-interacting factor-like (BdPIL) proteins. **(A)** Yeast two-hybrid analysis of BdPIL1 (Bradi1g13980) and BdPIL3 (Bradi2g11100) with Brachypodium phytochromes. BdAN and BdAC, N- and C-terminal domains of *B. distachyon* phytochrome A (BdphyA); BdBN and BdBC, N- and C- terminal domains of *B. distachyon* phytochrome B (BdphyB). Yeast cells were grown on non-selective (DDO) and selective (QDO/X/A) media. P53 × T and Lam × T were included as positive and negative controls, respectively. **(B)** β-galactosidase assay to quantify the interactions shown in A. Data represent the means ± SD from three independent replicates, and significant difference is indicated by ** (*P* < 0.01, Tukey’s test). **(C)** *In vitro* protein–protein interaction analysis between BdPILs and phytochromes. Pr and Pfr forms of full-length *Avena sativa* phyA (AsphyA), BdphyA, or *Arabidopsis thaliana* phyB (AtphyB) was incubated with GST/strep-tagged BdPIL1 or BdPIL3. Glutathione bead-bound proteins (output) were analyzed by western blotting. **(D)** Expression analysis of *BdPIL1* and *BdPIL3* in the absence and presence of light. Five-day-old dark-grown seedlings of Brachypodium (inbred line Bd21-3) were either kept in the dark or transferred to white light (WL, 100 μmol⋅m^–2^⋅s^–1^) for 1 and 4 h before harvesting for qRT-PCR analysis. **(E)** Expression analysis of *BdPIL1* and *BdPIL3* in different tissues of Brachypodium plants. For the qRT-PCR analysis, *BdUBC18* expression level was used for data normalization, and the relative expression levels were estimated by setting the transcript level in dark-grown **(D)** or root **(E)** samples as 1. Data represent the means ± SD of three independent biological replicates.

To determine whether BdPIL1 and BdPIL3 are genuine PIFs, we first verified their interaction with phytochromes by yeast two-hybrid (Y2H) assays. Using N- and C-terminal domains of *B. distachyon* phytochromes A (BdphyA) and B (BdphyB) as baits, we observed the interaction of both BdPIL1 and BdPIL3 with C-domains of both phytochromes (Figures 1A,B). It was also noted that the interactions of BdPILs with the C-domain of BdphyB was stronger than that with the C-domain of BdphyA. In addition, we performed Y2H assays using full-length BdphyA and BdphyB in the presence of phycocyanobilin (PCB) as chromophore, and observed that BdPIL1 and BdPIL3 interacted with both BdphyA and BdphyB under red light condition, but not in the dark (Supplementary Figure 2). These results suggest that both BdPIL1 and BdPIL3 interact with Brachypodium phytochromes in a Pfr-specific manner.

To verify the Pfr-specific interaction further, we performed *in vitro* pull-down assays using BdPIL1 and BdPIL3 proteins with phytochromes. For this, we initially expressed three phytochromes found in Brachypodium [BdphyA (Bradi1g10520), BdphyB (Bradi1g64360), and BdphyC (Bradi1g08400)] using the *Pichia pastoris* protein expression system as reported previously (Han et al., 2019). However, BdphyB and BdphyC were not expressed in this system, and only BdphyA could be expressed and purified in a sufficient amount for spectroscopic analysis (Supplementary Figures 3A,B). Thus, we used *A. thaliana* phyB (AtphyB), as well as *Avena sativa* phyA (AsphyA), for the pull-down assays. In the case of BdPIL1 and BdPIL3, recombinant proteins with the fusion of glutathione S-transferase (GST) were prepared using an *E. coli* protein expression system (Supplementary Figure 3C). The pull-down assays revealed the Pfr-specific interaction of BdPIL1 and BdPIL3 with all three phytochromes (Figure 1C). Overall, these results suggest that the conserved APB motifs in BdPIL1 and BdPIL3 function similarly to Arabidopsis PIF1 and PIF3 for the interaction with both phyA and phyB in an active form (Pfr)-specific manner.

### Expression of *BdPIL1* and *BdPIL3* in Brachypodium

To characterize BdPIL1 and BdPIL3 in Brachypodium, we determined whether their gene expression was regulated by light. Both *BdPIL1* and *BdPIL3* were upregulated under light conditions, compared with the transcript level in the dark (Figure 1D). It was also noted that the expression level of light-induced *BdPIL3* was higher than that of *BdPIL1*. We then analyzed which wavelengths of light were effective for the expression of *BdPIL1* and *BdPIL3*, and found that the expression of both genes increased under R and white light, but not by blue light (Supplementary Figure 4). Furthermore, the expression of *BdPIL1* and *BdPIL3* was also induced by FR light, but lesser than that induced by R light.

To understand the functions of BdPIL1 and BdPIL3 in Brachypodium, we also analyzed the expression of *BdPIL1* and *BdPIL3* in different tissues such as the root, stem, leaf, and flower. Both *BdPIL1* and *BdPIL3* were upregulated in flowers compared with their expression levels in roots (Figure 1E), suggesting their roles in flowering regulation. In addition, the expression of *BdPIL3* was increased in the stem and leaf tissues, indicating its possible role in the regulation of leaf and stem growth.

### Photoresponses of Transgenic Brachypodium Seedlings With the RNA Interference Constructs of *BdPIL1* and *BdPIL3*

To investigate the roles of BdPIL1 and BdPIL3 in Brachypodium, we used the RNAi approach with the expectation of hypersensitive photoresponses, because PIFs are known as negative regulators of phytochrome signaling in Arabidopsis. For this, we used Bd21-3 inbred line of Brachypodium to generate transgenic plants harboring the RNAi constructs of *BdPIL1* and *BdPIL3* (Supplementary Figure 5 and Supplementary Tables 2, 3). Among the generated homozygous lines (designated as *BdPIL1*/RNAi and *BdPIL3*/RNAi), we selected two independent lines exhibiting the most decreased expression of *BdPIL1* (1–5 and 9–20) and *BdPIL3* (4–10 and 5–16) for further analyses (Supplementary Figures 6A,B). To verify specific RNAi-suppression, we also analyzed the expression of the five *BdPIL* genes in the plants of both RNAi lines and found the specific suppression of *BdPIL1* and *BdPIL3* in the *BdPIL1*/RNAi and *BdPIL3*/RNAi plants, respectively (Supplementary Figure 6C).

**FIGURE 2:**
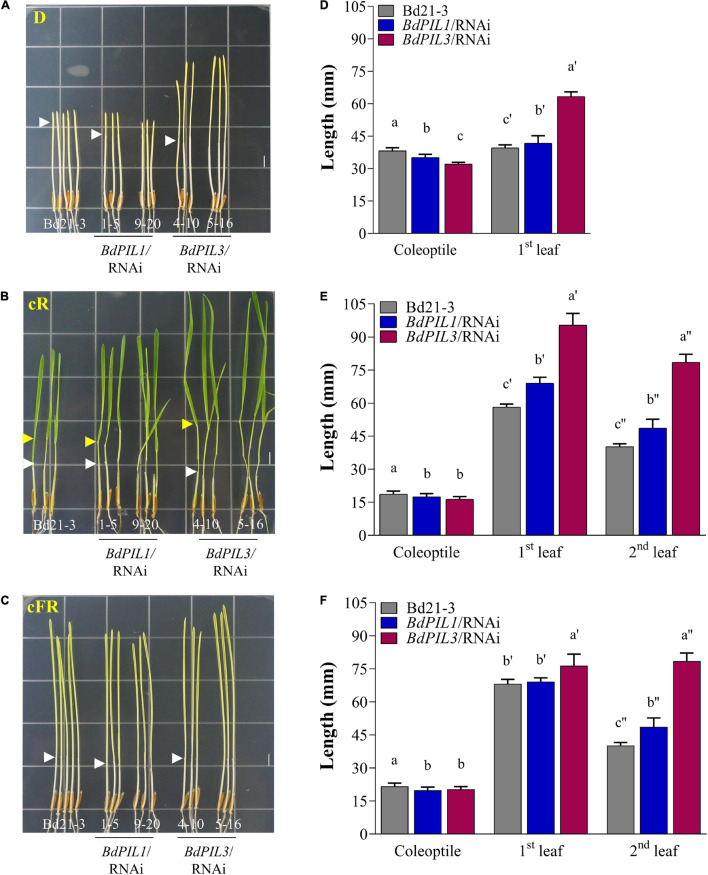
Photoresponses of transgenic Brachypodium plants harboring RNAi constructs of *BdPIL1* and *BdPIL3* to red and far-red light. **(A–C)** Representative seedlings grown in the dark (D), under continuous red (cR, 20 μmol⋅m^–2^⋅s^–1^), or continuous far-red (cFR, 20 μmol⋅m^–2^⋅s^–1^) light. White and yellow arrowheads indicate the ends of coleoptiles and the lamina joints of second leaves, respectively. Scale bar, 5 mm. **(D–F)** The lengths of coleoptile, first and second leaves of 6-day-old seedlings measured using ImageJ program. Data represent the means ± SD (*n* ≥ 30). Different letters represent significantly different means (*P* < 0.05, Duncan’s multiple range test).

Then, we determined the photoresponses of the transgenic plants cultivated under continuous red (cR) or continuous far-red (cFR) light. In the dark, the seedlings of both RNAi lines exhibited decreased length of coleoptiles but increased length of the first leaves (Figures 2A,D). Compared with the control plant (Bd21-3), the differences in the lengths of the coleoptiles and first leaves were higher in the *BdPIL3*/RNAi plants than in the *BdPIL1*/RNAi plants. Similar results were observed under cR and cFR light conditions (Figures 2B,C,E,F). The longest first and second leaves were observed in the *BdPIL3*/RNAi plants grown under cR light (Figures 2B,E). In monocots, exposure of seedlings to light inhibits the elongated growth of coleoptiles and causes leaves emerged from coleoptiles (Takano et al., 2009). Thus, the shorter coleoptiles and longer leaves in both RNAi lines suggest that BdPIL1 and BdPIL3 play negative roles in seedling development in response to light.

### Phenotypes of *BdPIL1*/RNAi and *BdPIL3*/RNAi Plants

**FIGURE 3:**
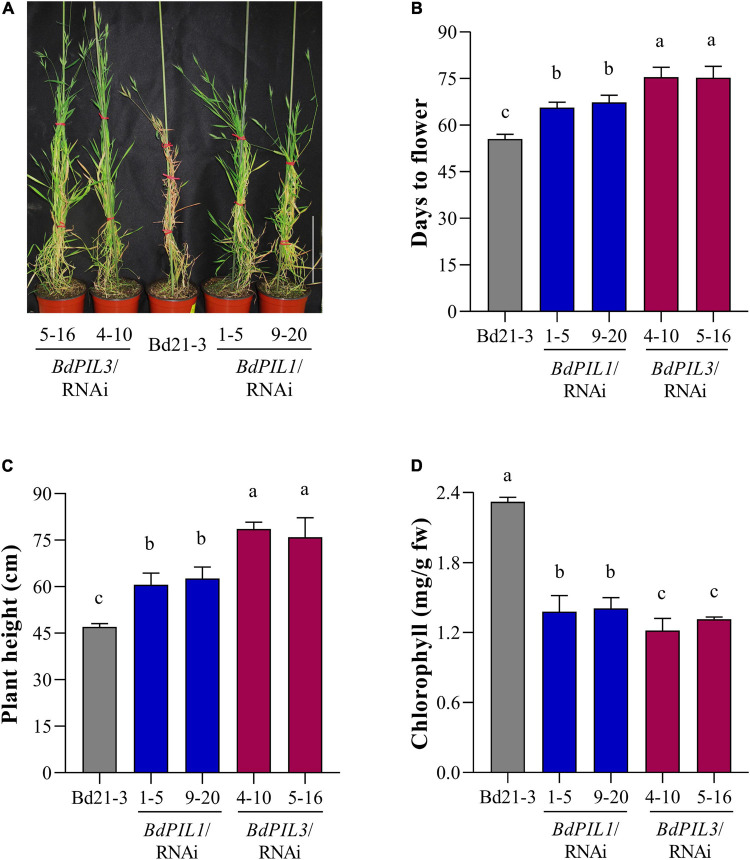
Phenotypic characterization of *BdPIL1*/RNAi and *BdPIL3*/RNAi plants. **(A)** Representative 80-day-old plants grown at 22°C under long day conditions (18-h light/6-h dark cycle). Wild-type Brachypodium (Bd21-3) plant served as a control. Scale bar, 20 cm. **(B)** Analysis of flowering time. The average flowering time was estimated from germination to the heading day. **(C)** Average plant heights of 4-month-old plants. **(D)** Chlorophyll content analysis using leaves of 8-week-old plants. Data represent the means ± SD (*n* ≥ 30 for **C,D**, *n* = 3 for **D**), and different letters represent significantly different means (*P* < 0.01, using Duncan’s multiple range test).

To determine the physiological functions of BdPIL1 and BdPIL3, we further analyzed the phenotypes of the transgenic plants. Among the phenotypes observed in both RNAi lines, late flowering was the most prominent (Figures 3A,B). In addition, the plants of both RNAi lines exhibited a longer vegetative growth period than Bd21-3, resulting in the tall phenotype (Figure 3C and Supplementary Figure 7A). Under the long-day conditions (18-h light/6-h dark cycle), the maximum height was attained approximately 60 days after germination by Bd21-3 plants, whereas the RNAi plants maintained the growth even after 80 days. It is also notable that the *BdPIL3*/RNAi plants exhibited more delayed flowering and taller phenotype than the *BdPIL1*/RNAi plants. As another apparent phenotypes in the plants of RNAi lines, pale green leaves were observed (Supplementary Figure 7B). Thus, we analyzed chlorophyll content in the leaves and found approximately two-fold lower total chlorophyll content in the RNAi plants than in Bd21-3 (Figure 3D). These findings suggest that BdPIL1 and BdPIL3 play roles in the transition to flowering and chlorophyll biosynthesis.

**FIGURE 4:**
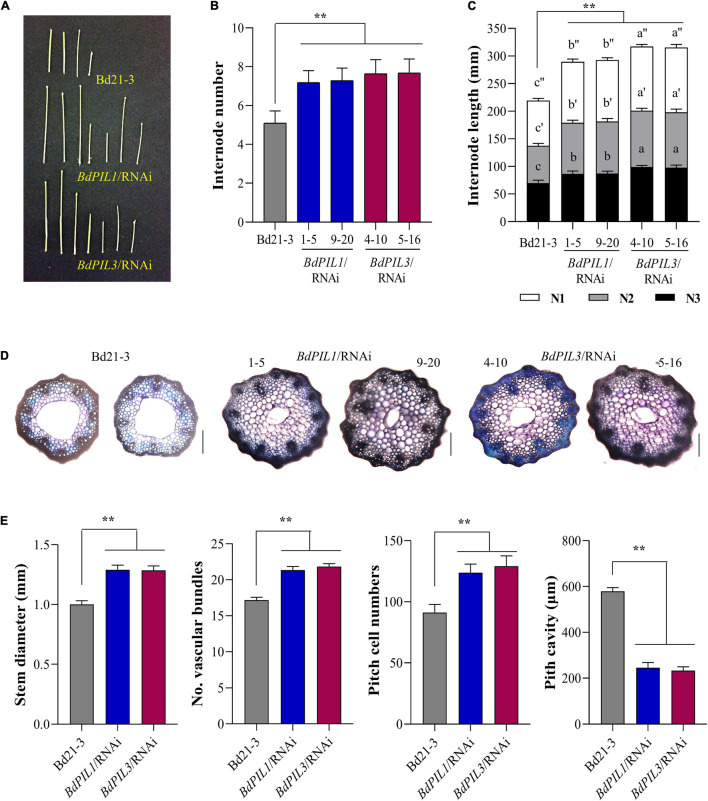
Stem internode elongation in the *BdPIL1/*RNAi and *BdPIL3/*RNAi plants. **(A)** Internodes obtained from 6-week-old plants. Scale bar, 3 cm. **(B)** Average number of internodes in the main stems of 4-month-old plants. **(C)** Average internode length of the three longest internodes (labeled as N1, N2, and N3 starting from the spikelet) in the main stem of 4-month-old plants. **(D)** Cross-sections and toluidine blue staining of the internode of the longest stem from 60-day-old plants. Scale bar, 250 μm. **(E)** Analysis of stem internodes. Stem diameter, number of vascular bundles and pitch cells, and pitch cavity of the longest internodes of 60-day-old plants were measured. Data represent the means ± SD (*n* ≥ 30 for **B**, **C**, *n* = 3 for **E**). Different letters in **(C)** represent significantly different means (*P* < 0.01, using Duncan’s multiple range test), and significant differences in comparison to Bd21-3 are indicated in **(E)** by ** (*P* < 0.01, Tukey’s test).

Moreover, to account for the tall phenotype, we investigated stems and found that the plants of both RNAi lines exhibited increased number of internodes compared with Bd21-3 (Figures 4A,B). Moreover, the lengths of the three longest internodes in the main stem were all longer than those in Bd21-3 (Figure 4C). Furthermore, we analyzed the anatomical features of the stems and observed increased stem diameter, more vascular bundles, and more pitch cells in the *BdPIL1*/RNAi and *BdPIL3*/RNAi plants (Figures 4D,E). By contrast, pitch cavity was reduced in the plants of both RNAi lines owing to the increased cell number (Figure 4E). These results suggest that the increased height of the both RNAi plants was largely dependent on the increased internode lengths because of the increase in the cell number.

### Regulation of Brachypodium Transcriptome by the RNA Interference-Suppression of *BdPIL1* and *BdPIL3*

**FIGURE 5:**
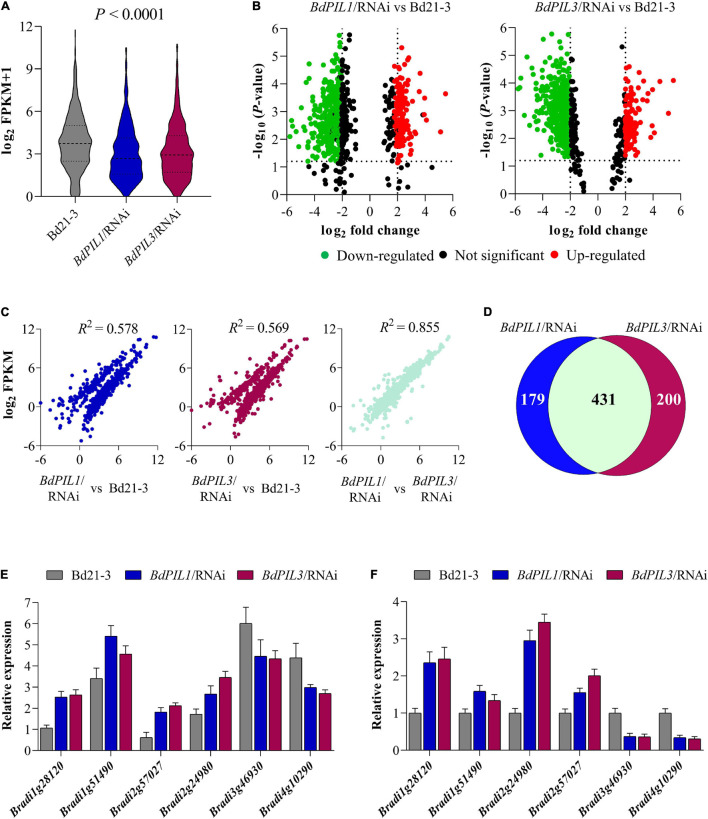
RNA-seq analysis of the RNAi plants versus Bd21-3 (wild-type) plants. **(A)** The range and distribution of FPKM values. **(B)** Volcano-plots of differentially expressed genes (DEGs) in *BdPIL1*/RNAi (left) and *BdPIL3/*RNAi (right) versus Bd21-3. Significantly downregulated and upregulated genes are shown in green and red dots, respectively. The vertical lines highlight log fold changes of ± 2, whereas the horizontal lines represent the values of significant differences in gene expression at *P* < 0.05. **(C)** Correlation analysis of the FPKM values. The scatter plots show the pairwise correlation among the libraries of three samples using Pearson’s correlation, and *R*^2^ values represent the correlation coefficients at *P* < 0.001. **(D)** Venn diagram to show the number of DEGs in *BdPIL1*/RNAi and *BdPIL3*/RNAi versus Bd21-3. The number of genes regulated by both *BdPIL1* and *BdPIL3* are indicated by the overlap between the two circles. **(E,F)** Transcriptome data of selected DEGs obtained from RNA-seq analysis **(E)** and analyzed using qRT-PCR **(F)** for data validation. Data represent the means ± SD of three independent biological replicates.

To examine how BdPIL1 and BdPIL3 regulate the observed phenotypes, we investigated the transcriptional changes associated with the RNAi-suppression of *BdPIL1* and *BdPIL3* using RNA-seq analysis (see Supplementary Data 1, 2). FPKM analysis revealed significant differences between the RNAi lines and wild-type (Bd21-3), exhibiting downregulation of many genes (Figure 5A). Compared with Bd21-3 (using fold change ≥ 2), 444 genes were downregulated, whereas 166 genes were upregulated in the *BdPIL1*/RNAi plant (Figure 5B). Similarly, 487 genes were downregulated, whereas 144 genes were upregulated in the *BdPIL3*/RNAi plant. Furthermore, the correlation analyses of the FPKM values revealed that the expression of genes in both *BdPIL1*/RNAi (*R*^2^ = 0.578) and *BdPIL3*/RNAi (*R*^2^ = 0.569) plants was significantly different from those in Bd21-3 (Figure 5C). By contrast, the correlation between the expression of genes in the *BdPIL1*/RNAi and *BdPIL3*/RNAi plants was noticeably high (*R*^2^ = 0.855), indicating largely overlapped differentially expressed genes (DEGs), i.e., 431 out of 610 and 631 DEGs in *BdPIL1*/RNAi and *BdPIL3*/RNAi, respectively (Figures 5C,D). Gene ontology (GO) analysis showed that the shared DEGs were involved in hormone signaling pathways such as auxin and gibberellin, cell wall and chlorophyll biosynthesis, and chloroplast development (Supplementary Figure 8 and Supplementary Data 3, 4). These data suggest that the functions of BdPIL1 and BdPIL3 overlap in Brachypodium, which are consistent with the similar phenotypes observed in the plants of both RNAi lines.

To validate the transcriptome data, we analyzed the expression of the following four upregulated and two downregulated genes using qRT-PCR: two small auxin upregulated RNA genes (*BdSAUR*; Bradi1g28120 and Bradi1g51490), two gibberellin 20 oxidase 2 genes (*BdGA20ox2*; Bradi2g57027 and Bradi2g24980), and two cell number regulator genes (*BdCNR*; Bradi3g46930 and Bradi4g10290). Similar to the RNA-seq results (Figure 5E), *BdSAUR* and *BdGA20ox2* genes were upregulated and *BdCNR* genes were downregulated in the plants of both RNAi lines (Figure 5F). Considering the function of *SAUR* and *GA20ox2* genes in Arabidopsis, such as the promotion of hypocotyl and internode elongation (Rieu et al., 2008; Dong et al., 2019), the upregulation of *BdSAUR* and *BdGA20ox2* was correlated with the elongated growth observed in both RNAi lines. Moreover, a previous study suggested that, when the expression of maize *CNR1* was suppressed, plant and organ size increased because of changes in cell number, but not cell size (Guo et al., 2010). In accordance, the present study found that the *BdCNR* genes were downregulated in the RNAi plants (Figure 5F), which might correlate with the increased cell number (Figure 4E). Collectively, these gene expression analyses verified the transcriptome data obtained in this study.

### Regulated Expression of Genes Related to Flowering and Chlorophyll Biosynthesis

**FIGURE 6:**
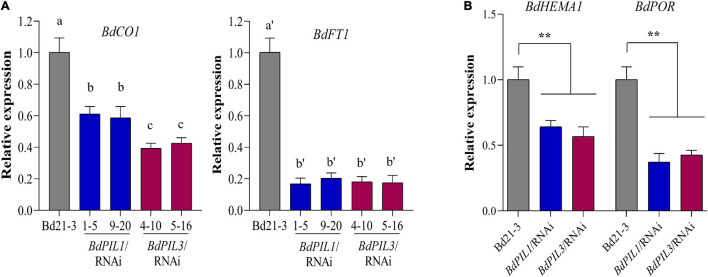
Gene expression analysis related to flowering and chlorophyll biosynthesis in the *BdPIL1/*RNAi and *BdPIL3/*RNAi plants. **(A)** qRT-PCR analysis of flowering genes, *BdCO1* (Bradi1g43671) and *BdFT1* (Bradi1g48830). RNA was extracted from 8-week-old plants, and the relative expression levels were estimated by setting the transcript level in Bd21-3 as 1, using *BdUBC18* expression levels for data normalization. Data represent the means ± SD of three independent replicates, and different letters represent significantly different means (*P* < 0.01, Duncan’s multiple range test). **(B)** qRT-PCR analysis of genes related to chlorophyll biosynthesis, *BdHEMA1* (*Bradi3g30160*) and *BdPOR* (*Bradi5g26230*). RNA was extracted from 5-day-old dark-grown seedlings. Significant changes in comparison to Bd21-3 are indicated by ** (*P* < 0.01, Tukey’s test).

Being late flowering and pale green leaves as apparent phenotypes of the *BdPIL1*/RNAi and *BdPIL3*/RNAi plants, we analyzed the expression of genes related to these phenotypes using RNA-seq data and qRT-PCR analysis. First, the transcriptome analysis revealed the decreased expression of genes that promote flowering, such as *CONSTANS1* (*BdCO1*/Bradi1g43671), *VERNALIZATION1* (*BdVRN1*/Bradi1g08340), and *CO-like* (*BdCOL2*/Bradi3g41500, *BdCOL14*/Bradi3g19011, and *BdCOL16*/Bradi3g57000) genes, and increased expression of genes that suppress flowering, such as *BdCOL4* (Bradi3g15490) and *BdCOL9* (Bradi1g43220) (Supplementary Figure 9A). Based on these results, we further analyzed the expression of *BdCO1* and *BdFT1* by qRT-PCR, because they play important roles in floral induction in Brachypodium (Lv et al., 2014; Feng et al., 2017; Qin et al., 2019). We compared the gene expression of *BdCO1* and *BdFT1* at the ZT20 and ZT8 stages, respectively, wherein the maximum expression was observed during the diurnal cycle (Supplementary Figure 10). The results showed that the expression of *BdCO1* and *BdFT1* was decreased in both RNAi lines (Figure 6A). Especially, *BdFT1* expression was strongly decreased to similar levels in both RNAi lines, whereas the decrease in *BdCO1* expression was more marked in the *BdPIL3*/RNAi plants than in the *BdPIL1*/RNAi plants. These findings were consistent with the extent of delay in flowering observed in the RNAi lines (Figures 3A,B). Thus, the RNAi-suppression of *BdPIL1* and *BdPIL3* repressed the expression of *BdCO1* and *BdFT1*, resulting in delayed flowering. Therefore, our results suggest that both BdPIL1 and BdPIL3 positively regulate floral induction.

Next, transcriptome analysis also revealed the downregulation of genes involved in chlorophyll biosynthesis, such as *BdChlH* (Bradi1g19220), *BdPBGD* (Bradi3g05160), *BdHEMA1* (Bradi3g30160), and *BdChlI* (Bradi1g49770) (Supplementary Figure 9B). Accordingly, we performed qRT-PCR analysis using dark-grown seedlings to investigate the expression of two important genes for chlorophyll biosynthesis, *BdHEMA1* (Bradi3g30160) and *BdPOR* (Bradi5g26230). The results showed significant suppression of these genes in the plants of both RNAi lines (Figure 6B), indicating that both BdPIL1 and BdPIL3 positively regulate chlorophyll biosynthesis. Together, our gene expression analyses were consistent with the results that the RNAi-suppression delays flowering and pale green leaves in Brachypodium plants.

### Regulated DNA-Binding Ability of BdPIL1 and BdPIL3 by Phytochromes

**FIGURE 7:**
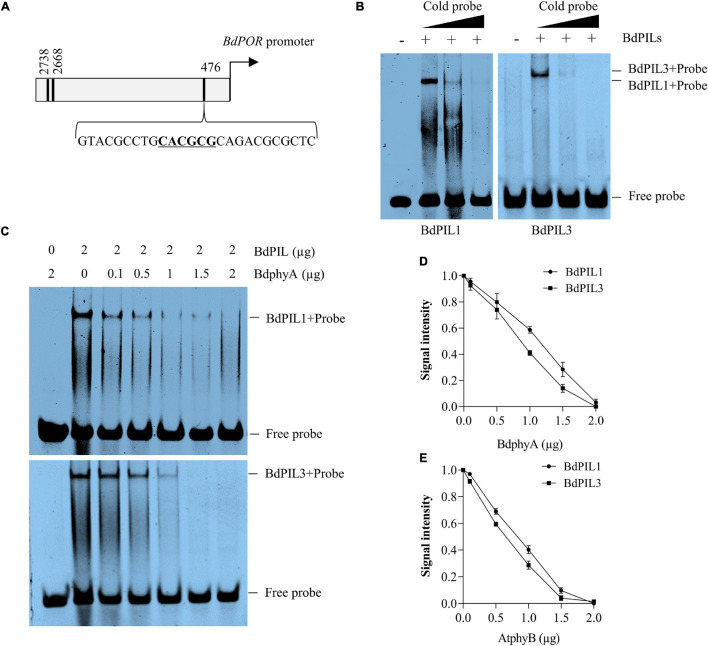
DNA-binding ability of BdPIL1 and BdPIL3 and the effects of phytochrome interactions. **(A)** A diagram depicting the promoter region of *BdPOR*. Locations of the predicted motifs (E- and N-boxes) for the binding of BdPILs are indicated with black lines. The third motif (underlined) was chosen as a probe. **(B)** DNA-binding ability of BdPIL1 and BdPIL3. Electrophoretic mobility shift assay (EMSA) was conducted by incubating BdPIL1 or BdPIL3 with ^32^P-labeled probe (labeled “+”). BSA was included as a negative control (labeled “–“). Competition assays were performed with 10× and 20× cold probes. **(C)** Effects of phytochrome interaction with BdPIL1 and BdPIL3 on their DNA-binding ability. BdPIL1 or BdPIL3 was incubated with the indicated concentrations of BdphyA (Pfr form) before EMSA. As negative controls, BdphyA with the ^32^P-labeled probe was included in the first lanes. **(D,E)** Quantification of DNA binding. Different amounts (0.1–2 μg) of Pfr forms of BdphyA **(D)** or AtphyB **(E)** were added to the reaction mixtures containing 2 μg of BdPIL1 or BdPIL3 before EMSA. DNA binding was estimated using the EMSA stains, and the signal from BdPIL-DNA complexes without phytochrome was assumed to be 1. Data represent the means ± SD of three independent replicates.

As PIFs are transcriptional factors with a bHLH motif, they bind to DNA. In Arabidopsis, it is known that phytochromes can inhibit the function of PIFs by preventing their DNA binding ability (Park et al., 2018; Favero, 2020). Therefore, we determined the DNA-binding ability of BdPIL1 and BdPIL3 using the promoter sequences of the putative target genes. As PIF proteins recognize G-, E-, and N-box motifs (Kim et al., 2016; Ji et al., 2019), we selected the following putative target genes containing G/E/N-box motifs in their promoter region: *BdPOR* (Bradi5g26230), *BdMIR156H* (LOC104794734), and *BdSAUR50* (Bradi1g51490). It is notable that there is only one protochlorophyllide reductase (*POR*) gene in Brachypodium and its expression was light-repressible, whereas the expression of *BdSAUR50* was induced under light conditions (Supplementary Figure 11). Using these promoter sequences containing one G/E/N box motif (Supplementary Table 1), we first verified the DNA-binding ability of both BdPIL1 and BdPIL3 by EMSA (Supplementary Figure 12A). Next, we further determined the effect of phytochrome interaction with BdPIL1 and BdPIL3 on their DNA-binding ability. For this, we first analyzed the DNA-binding ability of BdPIL1 in the presence of Pr and Pfr forms of BdphyA, and found that the DNA-binding was inhibited by the active Pfr form, but not by the inactive Pr form (Supplementary Figure 12B). Then, further analysis showed that both BdPIL1 and BdPIL3 bound to the promoter of *BdPOR* (Figures 7A,B), but the DNA binding was inhibited in the presence of BdphyA (Figures 7C,D). It is also notable that BdphyA inhibited the DNA binding of BdPIL3 more efficiently than that of BdPIL1. Moreover, BdphyA also prevented the binding of BdPIL1 and BdPIL3 to the promoter of *BdSAUR50* (Supplementary Figure 13), and AtphyB also showed similar inhibitory effects on the DNA-binding ability of BdPIL1 and BdPIL3 (Figure 7E and Supplementary Figure 14). Overall, these findings elucidate a molecular mechanism underlying the regulation of BdPILs by phytochromes in Brachypodium, i.e., sequestration, wherein phytochromes interact with BdPILs and prevent their binding to the promoters of the target genes.

## Discussion

Phytochrome-interacting factors, the primary signaling partners of phytochromes, regulate various aspects of plant growth and development in response to environmental cues, such as light and temperature (Paik et al., 2017; Pham et al., 2018). In this study, we performed the functional characterization of two PIFs in Brachypodium. Using phylogenetic and sequence analyses, five Brachypodium PIF-like (*BdPIL*) genes have been identified, among which two BdPILs homologous to Arabidopsis PIF1 and PIF3 were selected for the functional characterization. Another Brachypodium PIF (Bradi5g21950) was not included in the phylogenetic analysis owing to its small size (198 aa) compared to other BdPILs (>400 aa). Initially, we verified BdPIL1 and BdPIL3 as genuine PIFs by demonstrating their interaction with phytochromes in a Pfr-specific manner (Figure 1C and Supplementary Figure 2). BdPIL1 and BdPIL3 interacted not only with BdphyA and BdphyB but also with AsphyA and AtphyB, suggesting the involvement of their functional APB motifs in the interaction with both phyA and phyB and conserved nature of the phytochrome-PIF signaling module in dicot and monocot plants.

The functions of BdPIL1 and BdPIL3 in Brachypodium were investigated using RNAi lines. Overall, both *BdPIL1*/RNAi and *BdPIL3*/RNAi plants exhibited similar phenotypes, including decreased coleoptile lengths, increased leaf growth, elongated internodes, increased number of internodes, wide internode diameters with increased pitch cell number, late flowering with increased plant height, and pale green leaves with reduced chlorophyll content (Figures 2–4 and Supplementary Figure 7). Some of these phenotypes are comparable to the phenotypes of *Oryza sativa* PIF-like (OsPIL)-knockdown or knockout rice plants. For example, among six OsPILs (OsPIL11 to OsPIL16) (Nakamura et al., 2007), the T-DNA insertion knockdown rice of *OsPIL13* exhibited a pale-green phenotype with the downregulation of several genes involved in chlorophyll biosynthesis (Sakuraba et al., 2017), and the CRISPR/Cas9-mediated knockout rice of *OsPIL15* increased the number of cells in the grains (Ji et al., 2019). Furthermore, rice and maize plants overexpressing PIFs exhibited phenotypes related to seedling growth, internode elongation, and chlorophyll biosynthesis (Todaka et al., 2012; Zhou et al., 2014; Sakuraba et al., 2017; Shi et al., 2017; Mo et al., 2020). Here, we also reported similar phenotypes, but along with the positive regulatory roles of BdPIL1 and BdPIL3 in floral induction, which might be correlated with the expression of both *BdPIL1* and *BdPIL3* in the flower tissues (Figure 1E). In Arabidopsis, the overexpression of genes encoding PIFs, such as *PIF3*, *PIF4*, *PIF5*, and *PIF7*, accelerated flowering, whereas the knockout mutants exhibited late flowering phenotypes (Galvão et al., 2015; Zhang et al., 2019). Thus, the knockdown of genes encoding BdPILs in Brachypodium and the knockout of genes encoding PIFs in Arabidopsis exhibited similar flowering phenotypes. Overall, these findings suggest the important roles of both BdPIL1 or BdPIL3 in various aspects of plant growth and development, especially in regulating internode growth, flowering, and chlorophyll biosynthesis. Additionally, it is also notable that we could not observe any difference in seed germination between *BdPIL1*/RNAi and other Brachypodium plants, although Arabidopsis PIF1 is well known to inhibit seed germination. In this regard, further studies will be necessary to elucidate which BdPIL(s) involve in Brachypodium seed germination.

This study revealed the light-inducible expression of *BdPIL1* and *BdPIL3* in Brachypodium, especially under R light and to a lesser extent under FR light (Figure 1D and Supplementary Figure 4), indicating the role of phytochromes in the expression of the genes encoding *BdPILs* in Brachypodium. However, the expression patterns of *BdPIL1* and *BdPIL3* were different from those of Arabidopsis *PIF1* and *PIF3*, which are not light-inducible. In the case of Arabidopsis, the mRNA level of *PIF1* did not increase under R and FR light, whereas that of *PIF3* decreased (Oh et al., 2020). By contrast, the mRNA levels of *PIF4*, *PIF5*, *PIF7*, and *PIF8* were increased under both R and FR light, and the expression of *PIF2* and *PIF6* was suppressed by R light, indicating that the functions of BdPIL1 and BdPIL3 in Brachypodium might not be the same as PIF1 and PIF3 in Arabidopsis, although they have sequence homology. Therefore, further investigations are necessary to elucidate the functional differences between BdPILs and Arabidopsis PIFs.

The transcriptome analysis with the *BdPIL1*/RNAi and *BdPIL3*/RNAi plants suggested functional redundancy of *BdPIL1* and *BdPIL3* (Figures 5C,D), which was verified by similar phenotypes observed in both RNAi lines. Further analyses of DEGs suggested that *BdCNRs*, *BdCO1*/*BdFT1*, and *BdHEMA1*/*BdPOR* were associated with the phenotypes, including elongated internodes, delayed flowering, and pale-green leaves, respectively (Figures 5F, 6). In addition, the plants of both RNAi lines exhibit increased expression of *BdSAUR* and *BdGA20ox2* genes (Figures 5E,F), which might correlate with the tall phenotype, because these genes play roles in promoting elongated growth (Rieu et al., 2008; Stortenbeker and Bemer, 2019). Previously, OsPIL13 and OsPIL14 have been shown to bind to the promoters of the genes, such as *OsPORB* (Sakuraba et al., 2017; Mo et al., 2020). Thus, we analyzed the DNA-binding ability of BdPIL1 and BdPIL3 with the promoter sequences of selected target genes, namely, *BdPOR*, *BdMIR156H*, and *BdSAUR50*, and suggested that BdPIL1 and BdPIL3 regulated the transcription of target genes by directly binding to the corresponding promoters (Figure 7 and Supplementary Figures 12, 13). These findings propose that BdPILs control the transcription of various genes for regulating plant growth and development by binding to the target promoters.

The interaction of photoactivated phytochromes results in the inactivation of PIFs. Previous studies have shown that the Pfr form of phytochromes inhibits the DNA-binding ability of Arabidopsis PIF1, PIF3, and PIF4 using *in vitro* gel-shift assays (Martínez-García et al., 2000; Huq and Quail, 2002; Huq et al., 2004). Furthermore, PIFs have been shown to be inactivated by sequential phosphorylation, ubiquitination, and 26S proteasome-mediated degradation in a manner dependent on their interaction with active phytochromes (Al-Sady et al., 2006; Shin et al., 2016). Thus, phytochromes negatively regulate the activity of PIFs by their degradation and sequestration to induce photomorphogenic responses in plants (Qiu et al., 2017; Park et al., 2018). In this study, we could not investigate the degradation of BdPILs in Brachypodium because of the lack of detection tools, such as BdPIL-specific antibodies. Rather, we determined the effects of phytochrome interaction on the DNA-binding ability of BdPILs and verified that the interaction of BdPIL1 and BdPIL3 with the Pfr forms of BdphyA and AtphyB inhibited their DNA-binding ability (Figures 7C–E and Supplementary Figures 13, 14). Thus, we hypothesize that the sequestration of BdPILs from their target promoters is mediated via the interaction with photoactivated phytochromes, which regulates the activities of BdPILs. However, the degradation of BdPILs may be necessary for long-term regulation, although the sequestration might be effective for short-term regulation. Therefore, further investigations are necessary to elucidate the molecular mechanisms of phytochromes in regulating the activity of BdPILs.

According to the function of PIFs in Arabidopsis, PIFs are active in the dark to maintain skotomorphogenic developmental processes, but they are inactivated under the light conditions that generate the Pfr forms of phytochromes. Thus, it is hypothesized how BdPILs regulate the expression of genes involved in photomorphogenesis (Supplementary Figure 15). For example, *BdPOR* is expressed in the dark to regulate skotomorphogenesis, but it is repressed under light conditions (Supplementary Figure 11), and BdPILs regulate its transcription by the binding to the promoter sequence (Figure 7B). Thus, BdPILs positively regulate the transcription of *BdPOR* in the dark, but the transcription is inhibited by the sequestration of BdPILs from the promoter region owing to their interaction with phytochromes under light conditions. These results are consistent with a previous report that Arabidopsis PIF1 bound to a G-box motif of *PORC* promoter for transcriptional activation (Moon et al., 2008). As another example, in Arabidopsis, PIF3 has been shown to act as a transcriptional repressor in the dark for the expression of *SAUR* genes, including *SAUR50*, by competitively binding to the promoters with TCP4, a member of the teosinte branched1, CYCLOIDEA, and PCF transcription factor family (Dong et al., 2019). With a rapid inactivation of PIF3 upon light exposure, TCP4 bound to the promoters of the *SAUR* genes for transcriptional activation. In this study, we verified the light-inducible expression of *BdSAUR50* (Supplementary Figure 11) and the increased *BdSAUR50* expression in both RNAi lines (Figures 5E,F). In addition, we verified the binding of BdPIL1 and BdPIL3 to the promoter sequence of *BdSAUR50* (Supplementary Figure 12) and that phytochromes inhibited the binding of BdPIL1 and BdPIL3 to the *BdSAUR50* promoter (Supplementary Figure 13). These results suggest that similar regulatory mechanisms may operate in both Arabidopsis and Brachypodium; BdPIL1 and BdPIL3 act as a transcriptional repressor for the expression of *BdSAUR50* in the dark, whereas the expression of *BdSAUR50* is induced under light conditions via the inactivation of BdPIL1 and BdPIL3 by phytochromes (Supplementary Figure 15). Collectively, this study provides a molecular mechanism underlying the regulation of BdPILs by phytochromes and the roles of BdPILs as transcriptional regulators for the growth and development in Brachypodium.

## Data Availability Statement

The datasets presented in this study can be found in online repository and Supplementary Material. The name of the repository and accession numbers can be found below: Korean Bioinformation Center (KOBIC; www.kobic.re.kr) and accession codes of KBRS20191011_0000024 to KBRS20191011_0000032.

## Author Contributions

QH, Y-JH, and J-IK designed the research, analyzed the data, and wrote the manuscript. QH and ST obtained and analyzed transgenic Brachypodium plants. QH, A-YS, S-YK, and J-IK performed the transcriptome analysis. QH, ST, J-YC, D-MC, and Y-JH performed all other experiments. All authors approved the manuscript.

## Conflict of Interest

The authors declare that the research was conducted in the absence of any commercial or financial relationships that could be construed as a potential conflict of interest.

## Publisher’s Note

All claims expressed in this article are solely those of the authors and do not necessarily represent those of their affiliated organizations, or those of the publisher, the editors and the reviewers. Any product that may be evaluated in this article, or claim that may be made by its manufacturer, is not guaranteed or endorsed by the publisher.

## Footnotes

1
www.kobic.re.kr
