# Supplementary material for: Suppression of Phytochrome-Interacting Factors Enhances Photoresponses of Seedlings and Delays Flowering With Increased Plant Height in Brachypodium distachyon
Source: Front Plant Sci. 2021 Sep 28;12:756795. doi: 10.3389/fpls.2021.756795 (PMC8505764; doi:10.3389/fpls.2021.756795)
Supplement: Supplementary file 1 [file Data_Sheet_1.zip › Supplementary Figures and Tables.PDF]

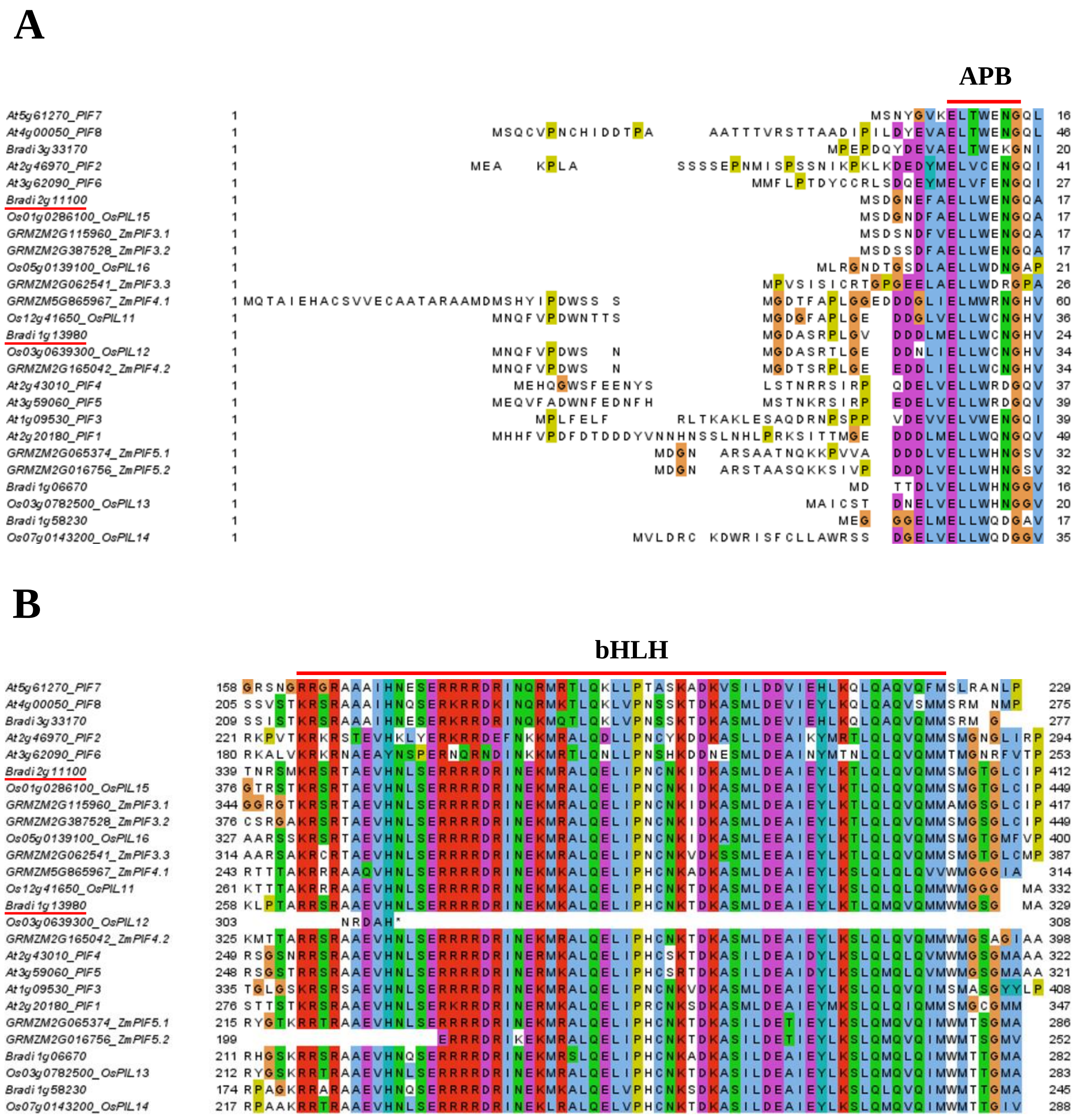
A
APB
B
bHLH

C

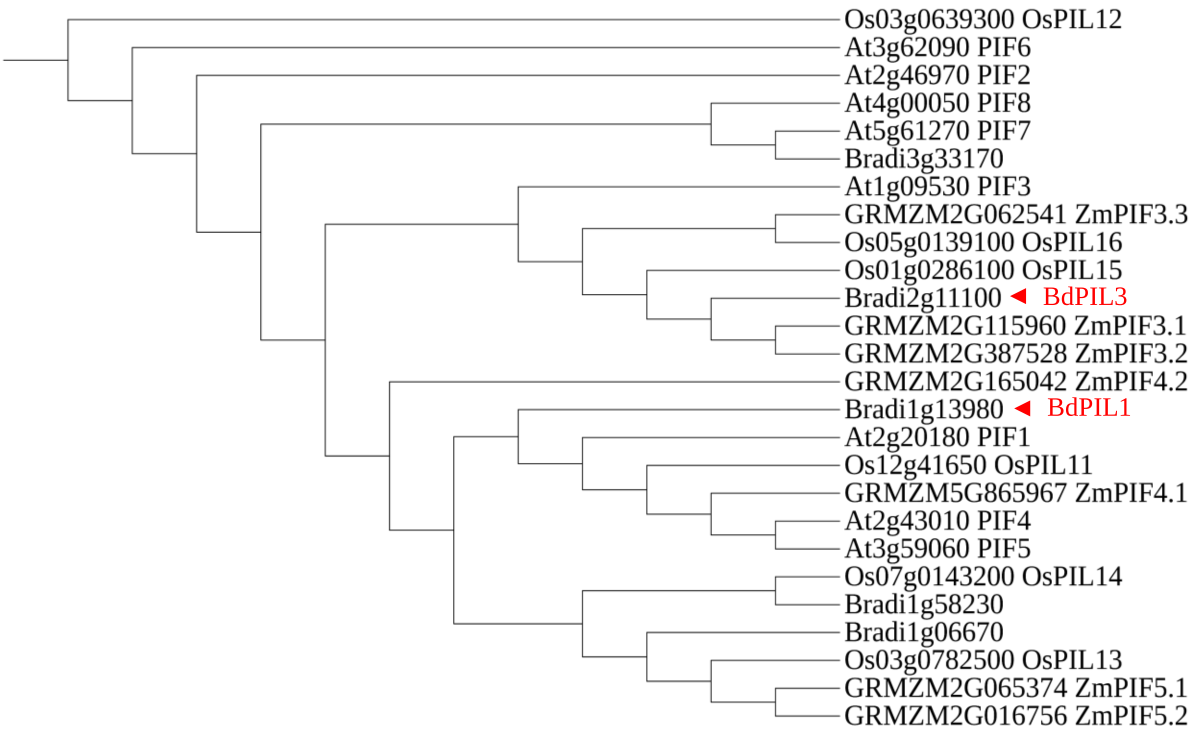

Supplementary Figure S1. Sequence and phylogenetic analyses of *Brachypodium distachyon* phytochrome-interacting factor-like (BdPIL) proteins.

(A-B) Alignment of amino acid sequences of active phytochrome-binding (APB) and basic helix-loop-helix (bHLH) motifs of phytochrome-interacting factors (PIFs) from *Arabidopsis thaliana* (8 genes; *PIF1* to *PIF8*), *Oryza sativa* (6 genes; *OsPIL11* to *OsPIL16*), *Zea mays* (7 genes; *ZmPIF3.1* to *ZmPIF5.2*), and *B. distachyon* (5 genes). The APB (A) and bHLH (B) motifs are shown and two Brachypodium PIFs used in this study are underlined in red. **(C)** Phylogenetic analysis. The cladogram was constructed based on the amino acid sequences using the maximum likelihood method (Bootstrap = 1,000). Phylogenetic tree was visualized using iTOL v6 (https://itol.embl.de). Bradi1g13980 (BdPIL1) and Bradi2g11100 (BdPIL3) were used in this study.

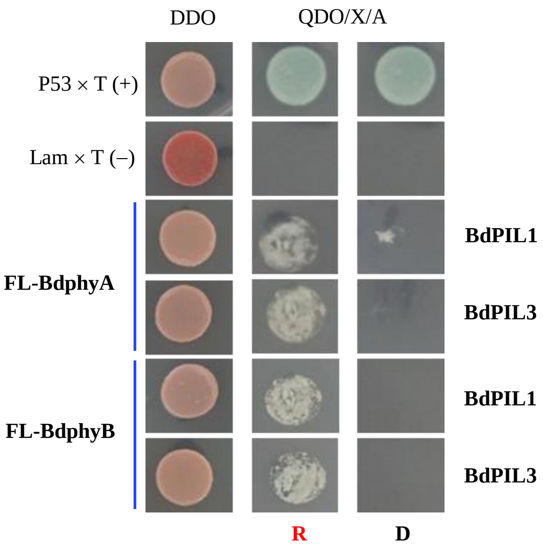

Supplementary Figure S2. Light-dependent interaction of BdPIL1 and BdPIL3 with full-length Brachypodium phytochromes A and B using yeast two-hybrid assays.
Full-lengths of *B. distachyon* phytochrome A (FL-BdphyA) and phytochrome B (FL-BdphyB) were fused to the DNA-binding domain and used as baits. Full-lengths of BdPIL1 (Bradi1g13980) and BdPIL3 (Bradi2g11100) were fused to the activation domain for the preys. Yeast cells co-expressing the indicated combinations of bait and prey were grown on non-selective (DDO) or selective (QDO/X/A) medium containing 20 µM of phycocyanobilin (PCB) as the chromophore for holo-phytochromes. The QDO/X/A plates were incubated under red light (R, 3 $\mu mol \cdot m^{-2} \cdot s^{-1}$) or in the dark (D) to represent the Pfr and Pr forms of phytochromes, respectively. P53 × T and Lam × T were included as the positive and negative controls, respectively.

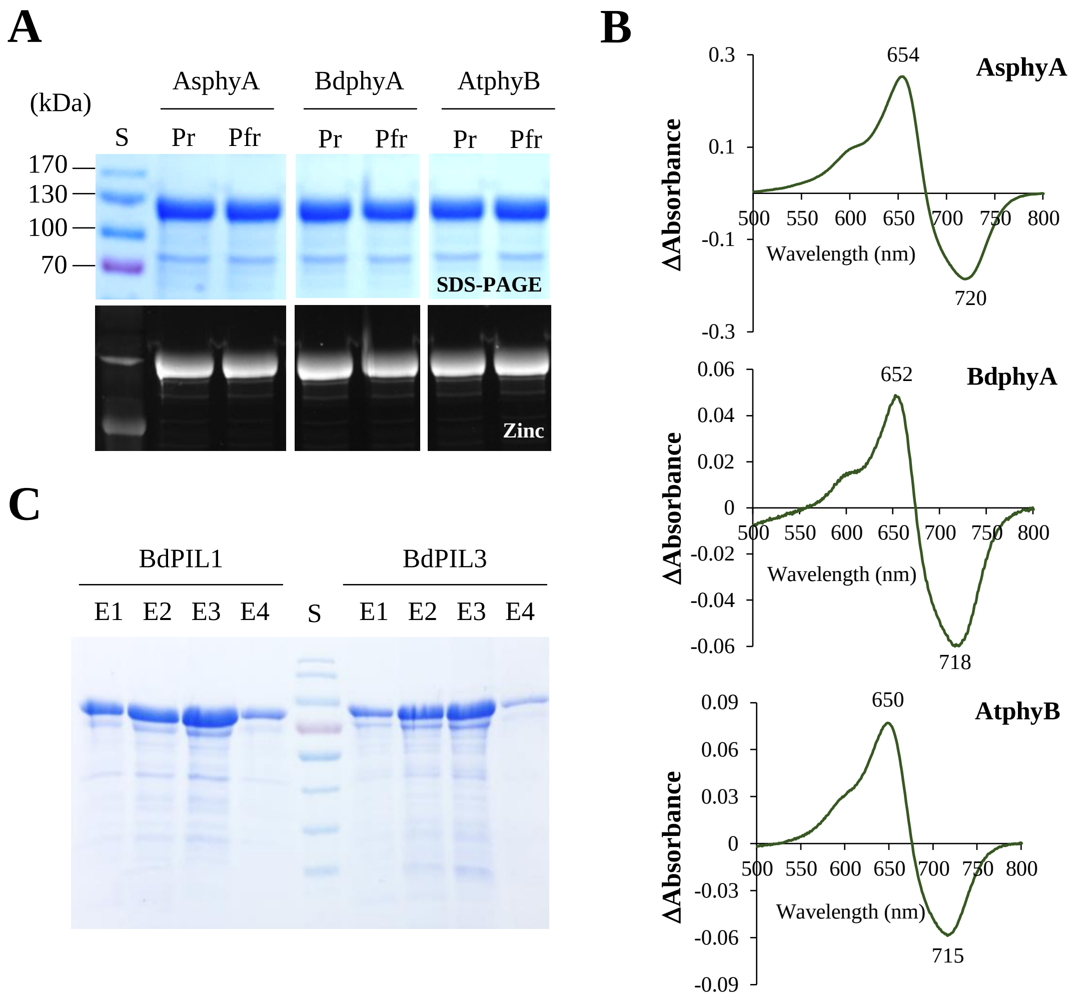

Supplementary Figure S3. Purified recombinant proteins used in this study.
(A) Purified phytochrome proteins. Full-length proteins of *Avena sativa* phyA (AsphyA), *Brachypodium distachyon* phyA (BdphyA), and *Arabidopsis thaliana* phyB (AtphyB) were expressed in the *Pichia pastoris* protein expression system and purified by streptavidin affinity chromatography. Coomassie-stained gels (SDS-PAGE) and zinc fluorescence images (zinc) are shown with PageRuler prestained protein ladder (lane S). **(B)** Difference spectra of the purified phytochromes. The difference spectra (ΔAbsorbance) were obtained by subtracting the Pfr absorption spectra from the Pr absorption spectra. Maximum and minimum absorbance peaks (λ_{max}) are labeled as numbers (nm) in the graphs. **(C)** Purified BdPIL1 and BdPIL3 proteins. The glutathione S-transferase/streptavidin (GST/strep)-tagged BdPIL1 (74.3 kDa) and BdPIL3 (85.8 kDa) proteins were expressed in *E. coli* and purified using streptavidin affinity chromatography. Lane S, PageRuler prestained protein ladder; lanes E1-E4, elution fractions of purified BdPIL1 (left) or BdPIL3 (right).

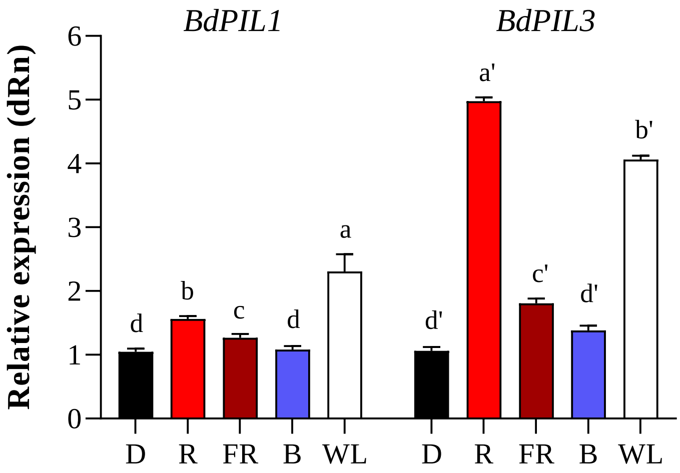

Supplementary Figure S4. Expression of *BdPIL1* and *BdPIL3* under different light conditions.

Five-day-old dark-grown seedlings of Brachypodium (inbred line Bd21-3) were either kept in the dark (D), transferred to red (R, 20 μmol·m^{-2}·s^{-1}), far-red (FR, 20 μmol·m^{-2}·s^{-1}), blue (B, 20 μmol·m^{-2}·s^{-1}), or white light (WL, 100 μmol·m^{-2}·s^{-1}). After incubation for 4 h, the seedlings were harvested for qRT-PCR analysis. The expression level of *BdUBC18* was used for data normalization, and the relative expression levels were estimated by setting the transcript level in dark-grown sample as 1. Data represent the means ± SD of three independent biological replicates, and different letters represent significantly different mean ($P < 0.05$ for *BdPIL1* and $P < 0.01$ for *BdPIL3*, Duncan's multiple range test).

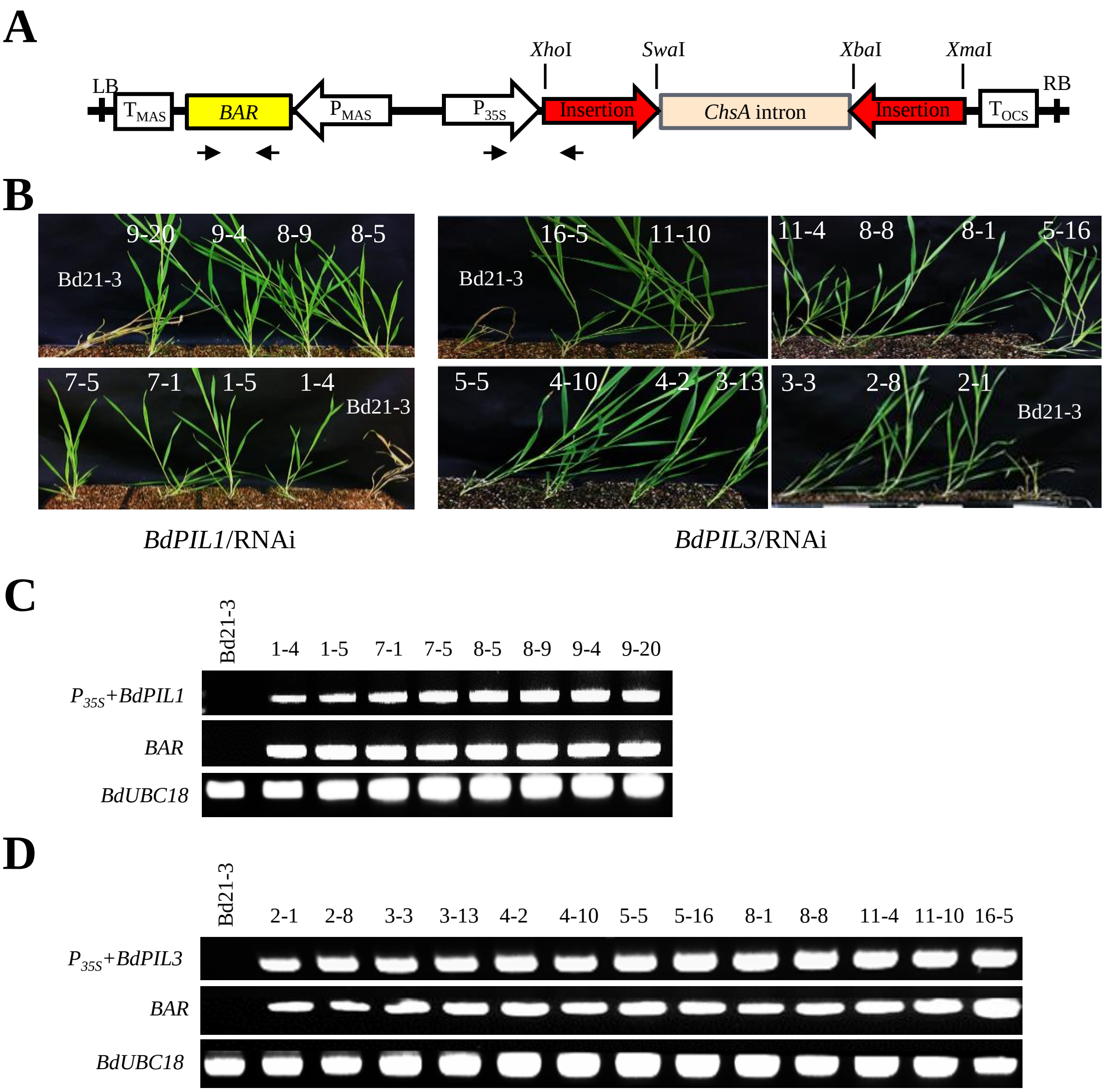

Supplementary Figure S5. Generation of transgenic Brachypodium plants expressing RNAi constructs of *BdPIL1* and *BdPIL3*.

(A) T-DNA region of the binary vector pFGC5941 harboring RNAi cassettes for *BdPIL1* or *BdPIL3*. LB and RB, left and right border sequences; P_{MAS}, mannopine synthase (*MAS*) promoter; *BAR*, phosphinotricin acetyltransferase gene; T_{MAS}, *MAS* terminator; P_{35S}, CaMV 35S promoter; *ChsA* intron, chalcone synthase A intron from *Petunia hybrida*; T_{OCS}, octopine synthase (*OCS*) terminator. Partial sequences of *BdPIL1* (68~550 bp) and *BdPIL3* (152~685 bp) were cloned into the vector in opposite orientations, separated by *ChsA* intron. The black arrows indicate the primers used for genomic PCR analysis. **(B)** Herbicide resistance assay. Numbers represent putative transgenic lines (*BdPIL1*/RNAi and *BdPIL3*/RNAi), and Bd21-3 was included as negative controls. Herbicide resistance was determined a week after 0.8% BASTA® treatment. **(C-D)** Genomic PCR analysis to verify the T-DNA integration of *BdPIL1* (C) and *BdPIL3* (D) in the transgenic Brachypodium plants. *BdUBC18* was shown as loading controls.

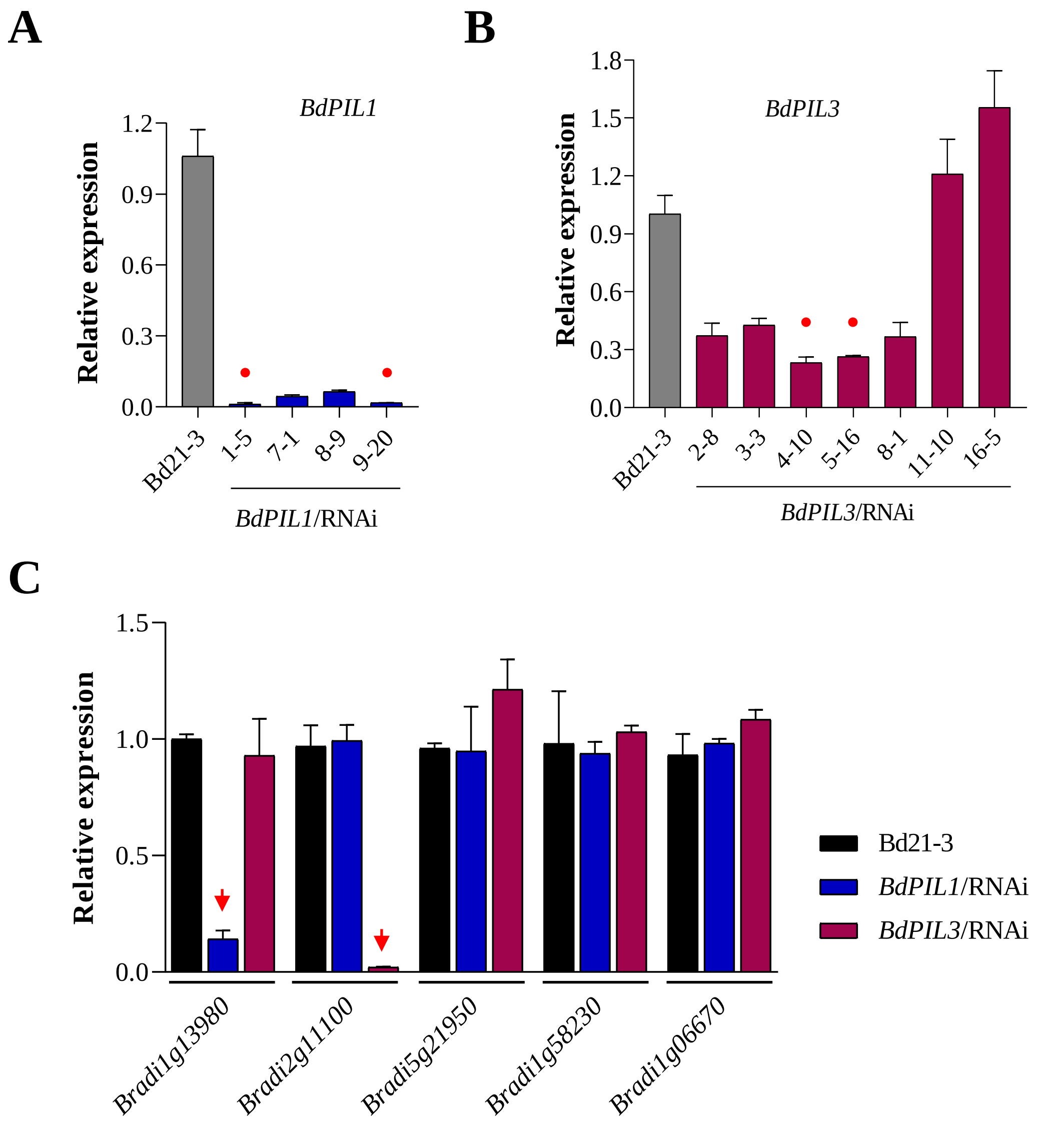

Supplementary Figure S6. Expression analysis of *BdPILs* in the transgenic plants with the RNAi constructs of *BdPIL1* and *BdPIL3*.

(A-B) Expression analysis of *BdPIL1* and *BdPIL3* to select transgenic lines with the corresponding RNAi constructs. Numbers represent independent homozygous transgenic lines of *BdPIL1*/RNAi and *BdPIL3*/RNAi. 5-d-old dark-grown seedlings were transferred to white light (100 $\mu mol \cdot m^{-2} \cdot s^{-1}$) for 4 h and harvested for qRT-PCR analysis. Red dots indicate the selected lines used for further analyses (1-5 and 9-20 for *BdPIL1*/RNAi; 4-10 and 5-16 for *BdPIL3*/RNAi). **(C)** Expression of five *BdPILs* in the *BdPIL1*/RNAi and *BdPIL3*/RNAi plants. Arrows indicate the suppression of *BdPIL1* and *BdPIL3* in the *BdPIL1*/RNAi and *BdPIL3*/RNAi plants, respectively. *BdUBC18* expression level was used for data normalization, and the relative expression levels were estimated by setting the transcript level in Bd21-3 as 1. Data represent the means ± SD of three independent biological replicates.

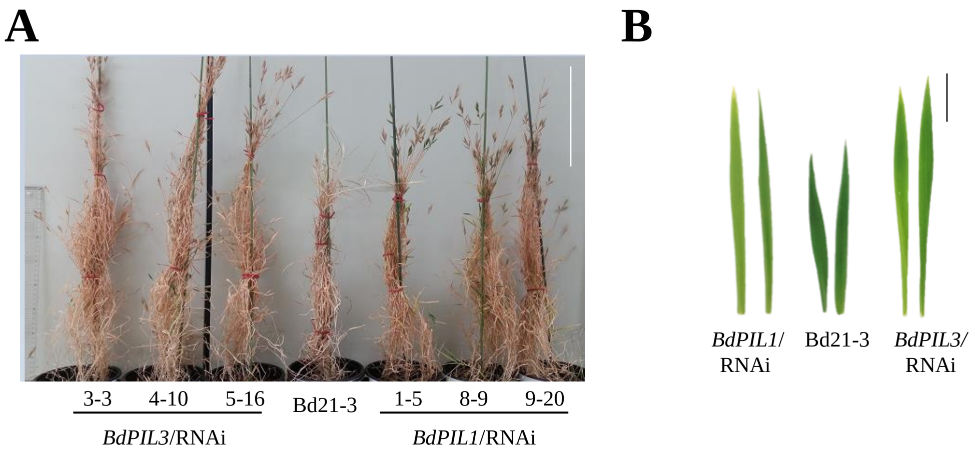

Supplementary Figure S7. Phenotypic characterization of *BdPIL1*/RNAi and *BdPIL3*/RNAi plants.
(A) Representative 5-month-old plants grown at 22°C under long day conditions (18-h light/6-h dark). Wild-type Brachypodium (Bd21-3) plant served as a control. Scale bar, 20 cm. **(B)** Representative leaves of 8-week-old Bd21-3 (wild-type) and the RNAi plants. Scale bar = 2 cm.

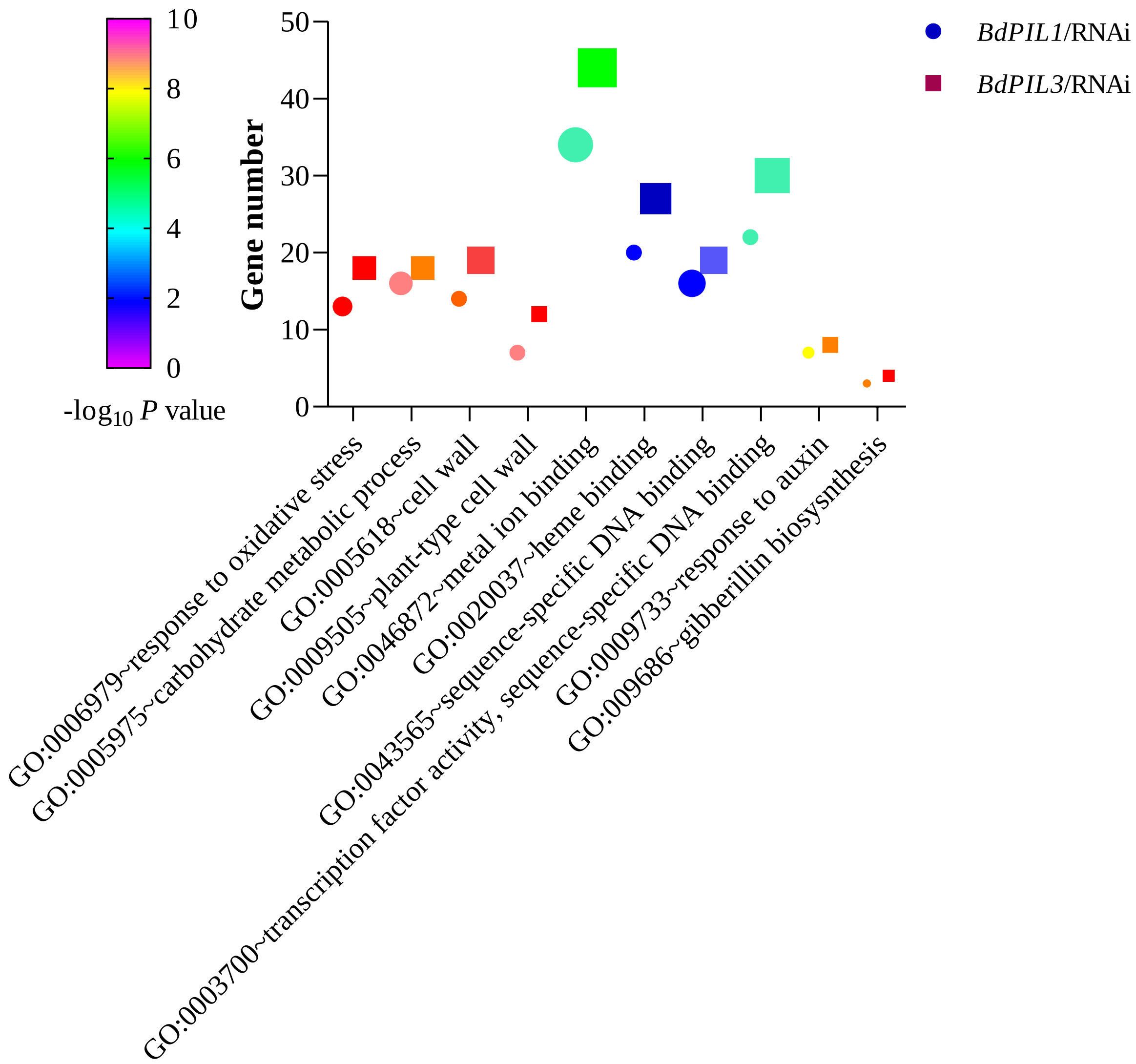

Supplementary Figure S8. Analysis of differentially expressed genes (DEGs) in the RNAi lines of *BdPIL1* and *BdPIL3*.

Using the RNA-seq data, gene ontology (GO) analysis of the 10 most popular groups showed large overlaps in the DEGs between *BdPIL1*/RNAi (circle) and *BdPIL3*/RNAi (square) plants. The sizes are proportionate to the number of genes in the groups, and different colors represent the *P* values. The GO analysis results were included as Supplementary Data 3 and 4.

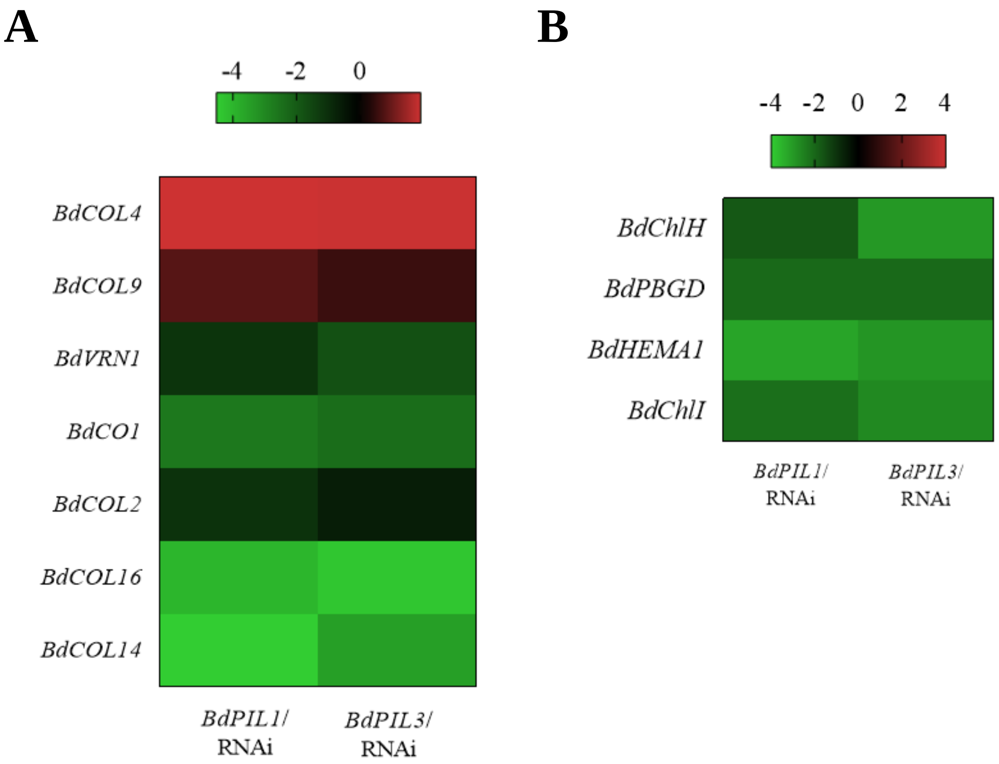

Supplementary Figure S9. RNA-seq data showing gene expression related to flowering and chlorophyll biosynthesis.
(A) A heatmap showing the expression patterns of genes involved in flowering, including *CONSTANS1* (BdCO1), *CONSTANS-LIKE* (*BdCOLs*), and *VERNALIZATION1* (*BdVRN1*). **(B)** A heatmap showing the expression patterns of genes involved in chlorophyll biosynthesis, including *BdHEMA1* (glutamyl-RNA reductase), *BdPBGD* (porphobilinogen deaminase), *BdChlI* and *BdChlH* (magnesium-chelatase subunits). The color of each gene is based on the log_2 fold change in the expression levels from RNA-seq analysis. The RNA-seq results were included as Supplementary Data 1 and 2.

A

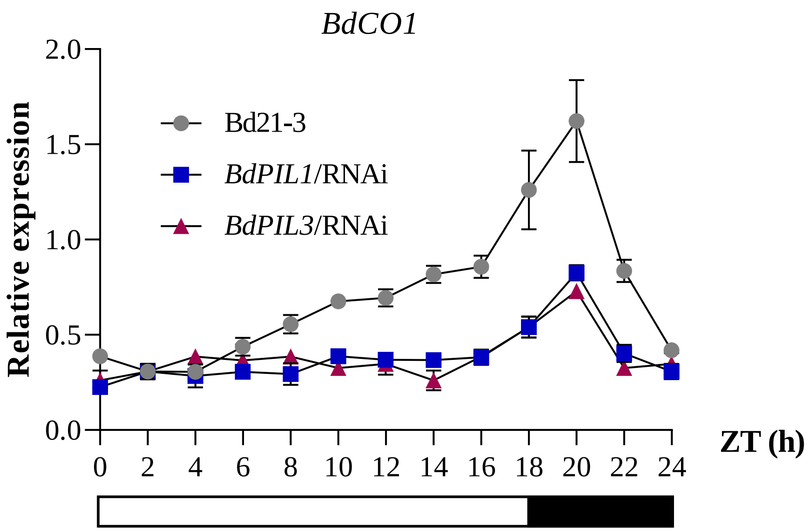

B

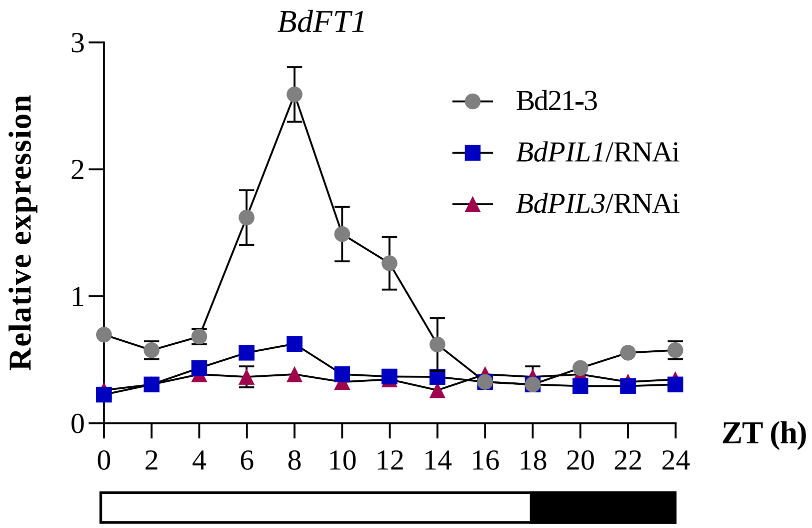

Supplementary Figure S10. Expression analysis of *BdCO1* (A) and *BdFT1* (B) under a long day cycle.

Brachypodium plants were grown for 8 weeks under long day conditions (18-h light/6-h dark cycle) and harvested for sampling at 2 h-intervals. The expression levels of *BdCO1* and *BdFT1* were quantified from the gel images of RT-PCR using ImageJ, and *BdUBC18* expression level were used for normalization.

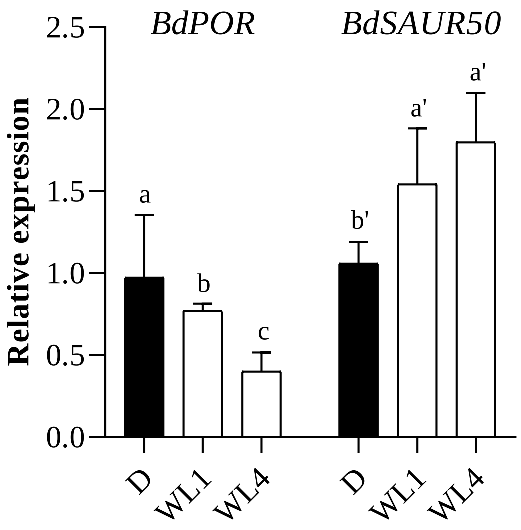

Supplementary Figure S11. Light-regulated expression of *BdPOR* and *BdSAUR50*. RNA was extracted from 5-d-old dark-grown (D) seedlings and those transferred to white light (WL, 100 μmol·m^{-2}·s^{-1}) for 1 or 4 h. The expression of *BdPOR* and *BdSAUR50* was then analyzed using qRT-PCR. *BdUBC18* expression level was used for data normalization, and the relative expression levels were estimated by setting the transcript level in D as 1. Data represent the means ± SD of three independent biological replicates, and different letters represent significantly different means (P < 0.01, Duncan's multiple range test).

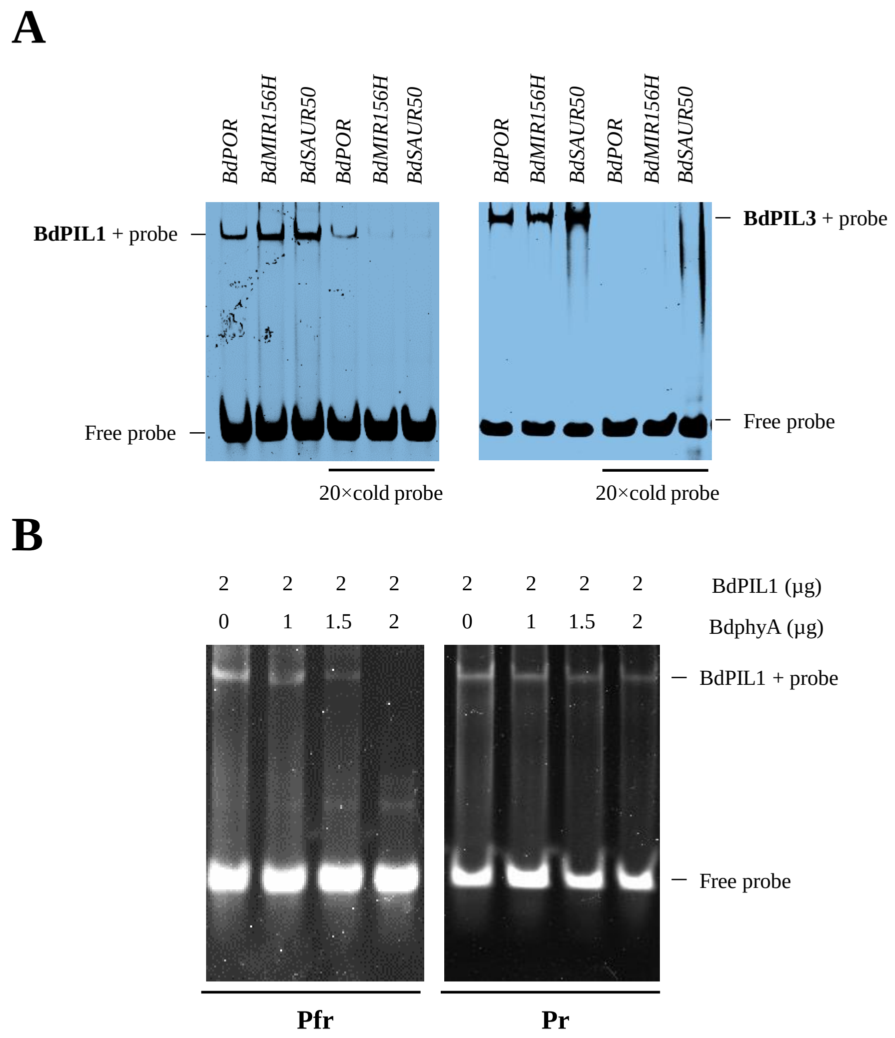

Supplementary Figure S12. Analysis of DNA-binding ability of BdPIL1 and BdPIL3. (A) DNA-binding assays of BdPIL1 and BdPIL3. Promoter sequences containing G-, E-, or N-box motifs were obtained from three putative target genes, *BdPOR* (Bradi5g26230), *BdMIR156H* (LOC104794734), and *BdSAUR50* (Bradi1g51490), and used as probes after 5′-end-labeling with ^{32}P-ATP. For the DNA-binding assays, 2 μg of purified BdPIL1 or BdPIL3 was incubated with 1 pmol of ^{32}P-labeled probes. Competition assays were also performed using 20× cold probes. **(B)** DNA-binding assays of BdPIL1 in the presence of Pr and Pfr forms of BdphyA. 2 μg of BdPIL1 was incubated with the indicated concentrations of BdphyA (Pfr or Pr) before electrophoretic mobility shift assay (EMSA). DNA bands were visualized using SYBR™ Green & SYPRO™ Ruby EMSA stains.

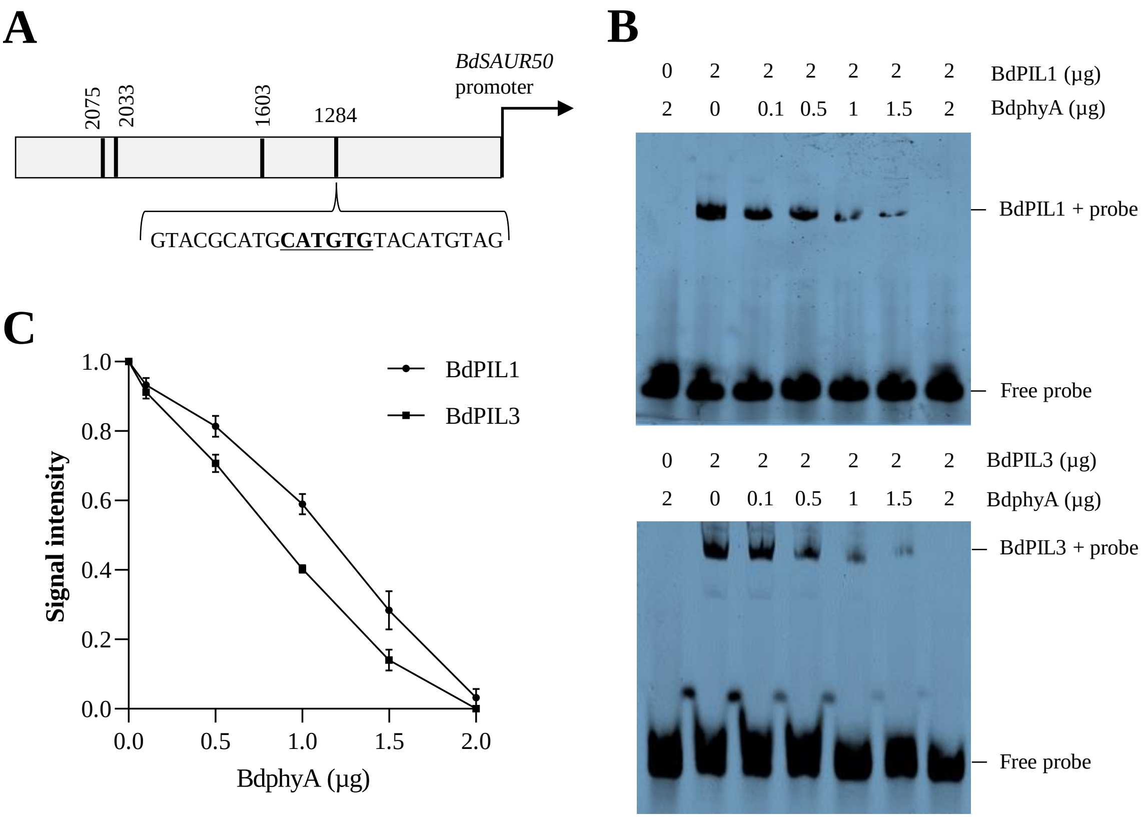

Supplementary Figure S13. Effects of phytochrome interaction with BdPIL1 and BdPIL3 on their DNA-binding ability.

(A) Diagram depicting the promoter region of *BdSAUR50*. The 3-kb upstream sequence of *BdSAUR50* was used for promoter analysis. Locations of the predicted four E-boxes for the binding of BdPILs are indicated with black lines. The sequence in the 4th motif was chosen as a probe for the DNA-binding assays. **(B)** DNA-binding ability of BdPIL1 and BdPIL3 in the presence of BdphyA. 2 µg of BdPIL1 (upper) or BdPIL3 (lower) was incubated with the indicated concentrations of BdphyA (Pfr form) before electrophoretic mobility shift assay (EMSA). As negative controls, 2 µg of BdphyA with 1 pmol of the ^{32}P-labeled probe was included in the first lanes. **(C)** Quantification of DNA binding. Different amounts (0.1-2 µg) of BdphyA (Pfr form) were added to the reaction mixtures containing 2 µg of BdPIL1 or BdPIL3 before EMSA. DNA-binding was estimated using SYBR™ Green & SYPRO™ Ruby EMSA stains, and the signal from BdPIL-DNA complexes without BdphyA was assumed to be 1. Data represent the means ± SD of three independent replicates.

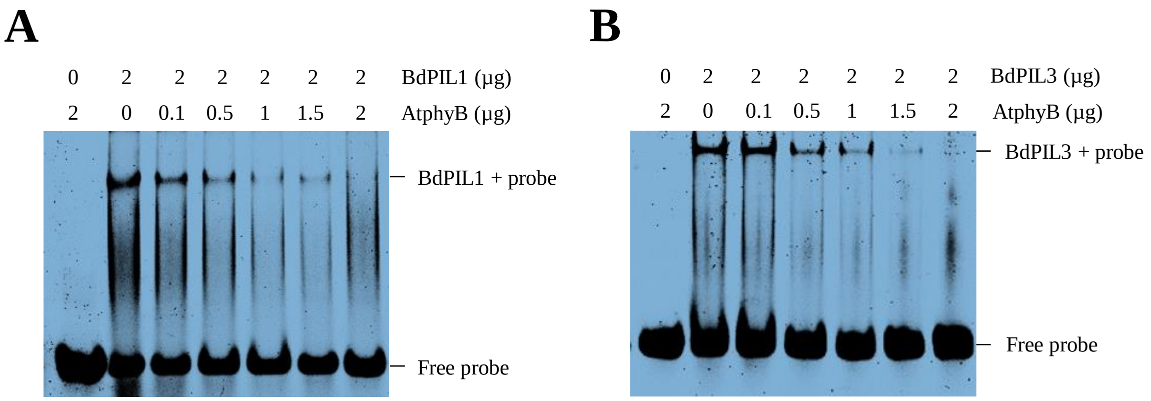

Supplementary Figure S14. Regulation of DNA binding of BdPIL1 and BdPIL3 by their interaction with Arabidopsis phytochrome B (AtphyB).

2 μg of BdPIL1 (**A**) or BdPIL3 (**B**) was incubated with the indicated concentrations of AtphyB (Pfr form) before electrophoretic mobility shift assay (EMSA) using ^{32}P-labeled *BdPOR* probe. As negative controls, 2 μg of AtphyB with 1 pmol of the probe was included in the first lanes.

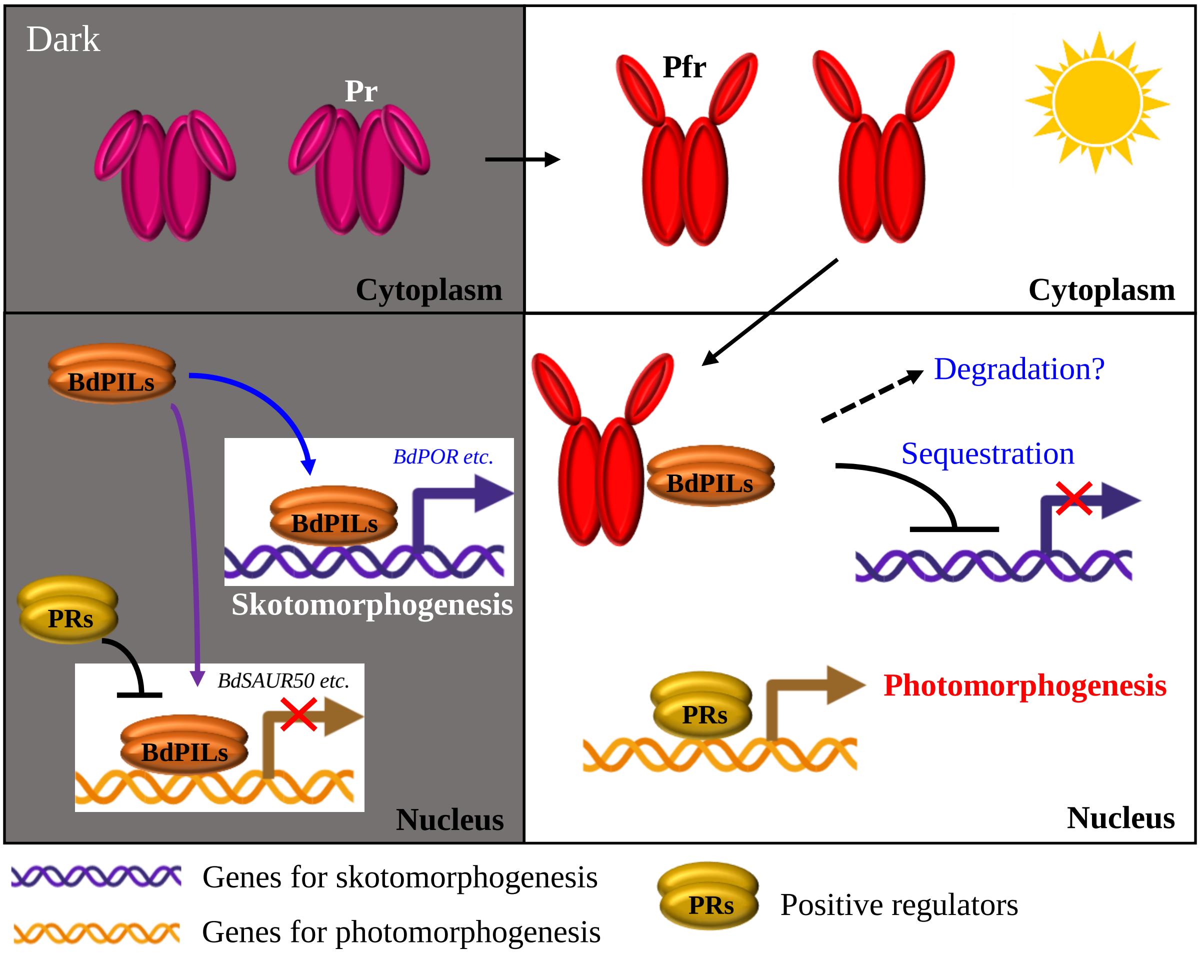

Supplementary Figure S15. A schematic model of the regulation of BdPIL1 and BdPIL3 by phytochromes in Brachypodium.

In the dark, phytochromes are synthesized as the Pr form, locating in the cytoplasm. In the nucleus, BdPIL1 and BdPIL3 bind to the target promoters: on the one hand, as transcriptional activators of the genes involved in skotomorphogenesis; on the other hand as transcriptional repressors of genes involved in photomorphogenesis, by competitively binding with the target promoters with positive regulators (PRs). Upon light exposure, photoactivated phytochromes (Pfr form) are localized into the nucleus and interact with BdPIL1 and BdPIL3, resulting in their inactivation by sequestration from the target promoters and possibly by inducing protein degradation. Thus, phytochromes induce the expression of light-responsive genes for photomorphogenesis via the functioning of PRs, while suppressing skotomorphogenesis.

Supplementary Table S1. Primers used in this study.

Oligo name	DNA sequence[a]
**For cloning into yeast two-hybrid (Y2H) vectors**	
Bradi1g13980 (BdPIL1)	5'-CGAATTCATGGGAGACGCATCCAGAC-3'
	5'-CCCCTCGAGGTATTCTGTTTGGATGCATCCC-3'
Bradi2g11100 (BdPIL3)	5'-CCGGATCCACCATGTCCGACGGCAAC-3'
	5'-CCCCTCGAGTCTGTTTAGCTTCCTTGTTCAG-3'
BdAN (N-domain)	5'-CCGGAATTCAATGTCTTCCTCAAGGCCTACTCAGTCTTCC-3'
	5'-CGCGGATCCATCAAGTTTTAGATCACCAATCTGG-3'
BdAC (C-domain)	5'-GGGTTTCATATGAGTTTGTCGTGGAGTGACTATGAG-3'
	5'-TCCCCCGGGTCATTGTCCAACTGCTGTTGGAGCCGAAGC-3'
BdBN (N-domain)	5'-GGGTTTCATATGATGGCCTCGGGCAGCCGCGCCAC-3'
	5'-TCCCCCGGGTAGTTCCCCAAGCTGAACCTGACC-3'
BdBC (C-domain)	5'-CCGGAATTCGGTCAGGTTCAGCTTGGGGAACTA-3'
	5'-CAACTGCAGTCAGCTCCTCTCCCTACTTTCCGCTTGCAG-3'
FL-BdphyA (Full-length)	5'-CGGGATCCATGTCTTCCTCAAGGCCTAC-3'
	5'-ATTTGCGGCCGCTCATTGTCCAACTGCTGTTG-3'
FL-BdphyB (Full-length)	5'-GGCCATGGAGGCCATGGCCTCGGGCAGCCGC-3'
	5'-TCCCCCCGGGTCAGCTCCTCTCCCTACTTTC-3'
**For cloning into pStrep (i.e., pGEX 4T-1 vector with strep-tag)**	
Bradi1g13980 (BdPIL1)	5'-CGCGAATCCATGGGAGACGCATCCAGAC-3'
	5'-CCCCTCGAGGTATTCTGTTTGGATGCATCCC-3'
Bradi2g11100 (BdPIL3)	5'-CGCGAATTCACCATGTCCGACGGCAAC-3'
	5'-CCCAGCGCTCTGTTTAGCTTCCTTGTTCAG-3'
**For cloning into pFGC5941 (i.e., a binary vector for RNAi)**	
Bradi1g13980 (68-550 bp)	5'-TCCCCCGGG*CTCGAG*ATGTGGTCATGCAGAGCCAGACTC-3'
	5'-GCTCTAGA*ATTTAAAT*CATTAATCCCGCTGCCCACCGCCGA-3'
Bradi2g11100 (152-685 bp)	5'-TCCCCCGGG*CTCGAG*ACATGATGGCGTTCACCAAGGCGGC-3'
	5'-GCTCTAGA*ATTTAAAT*CGCTCGTCGTCTGGATAACCGTG-3'
**For genomic PCR analysis**	
35S promoter	5′-CTATCCTTCGCAAGACCCTTC-3′ (forward)
Bradi1g13980	5′-CTCTCGGAGTAGTCCTCGGTGC-3′ (reverse)
Bradi2g11100	5′-GCCGACAAGCCCTGGATTTCC-3′ (reverse)
BAR	5′-CTACCATGAGCCCAGAACGACG-3′ (forward)
	5′-CTGCCAGAAACCCACGTCATGCCAGTTC-3′ (reverse)
**For real-time PCR analysis**	
Bradi1g13980 (*BdPIL1*)	5'-CAGGTGCAGGTGCAGCAC-3'
	5'-TCCGCTCGGAGAGGTTGT-3'
Bradi2g11100 (*BdPIL3*)	5'-GCTGTGGGAGAATGGGCAGGCG-3'
	5'-GGTGAGAGCCGACAAGCCCTGG-3'
Bradi4g00660 (*BdUBC18*)	5'-TGGAGGCACCTCAGGTCATTTTC-3'
	5'-GTTGCTTTGCTGGCGAGCTAGAC-3'

Bradi1g28120 (*BdSAUR36*)	5'-AGCAGAGCACTACCACAAGT-3'
	5'-GACGTATTCCATCACCGCCA-3'
Bradi1g51490 (*BdSAUR50*)	5'-CTCCAGTCCAATCCACCAGC-3'
	5'-ACATACACCGCGAAGTGGC-3'
Bradi2g24980 (*BdGA20ox2-1*)	5'-ATGCGGTGCAACTACTACCC-3'
	5'-TGGGGGTTTTCAGGAATCGG-3'
Bradi2g57027 (*BdGA20ox2-2*)	5'-GGTACAAGAGCTGCCTCCAC-3'
	5'-TTTTCGGCTTGAGACCCACA-3'
Bradi3g46930 (*BdCNR2*)	5'-CATCATGCTCCTCACCGGAT-3'
	5'-CTGCCTCTCCATGTTAGCGT-3'
Bradi4g10290 (*BdCNR13*)	5'-CTAGTTAGCCAGGCTGCCAT-3'
	5'-AAGGAGATCTCGCTCTGCAC-3'
Bradi1g43671 (*BdCO1*)	5'-TGATAGCCACATCTACAACAACC-3'
	5'-TGGAATCTGTGTAAGCACTGAC-3'
Bradi1g48830 (*BdFT1*)	5'-CATCACAATCCATGGGCCCATACAG-3'
	5'-GCAGTCGCCGTCATTTACATGTGTG-3'
Bradi3g30160 (*BdHEMA1*)	5'-GCCTGTGTGTCTGATGTGGA-3'
	5'-GCGAACTCAGGGGTAAGTGG-3'
Bradi5g26230 (*BdPOR*)	5'-AGCCTACAAGGACAGCAAGG-3'
	5'-AACCCCAGACTTCGTCAAGC-3'
**For probes used in EMSA**[b]	
BdPOR (Bradi5g26230)	5'-CACGTACGCCTG*CACGCG*CAGACGCGCTCCATCGATTATACTACTTCCTCCGGCCGGAAT-3'
	5'-ATTCCGGCCGGAGGAAGTAGTATAATCGATGGAGCGCGTCTGCGCGTGCAGGCGTACGTG-3'
BdMIR156H	5'-TTGGTAATAGAAGTTGTTTGGTCGCACCATTGGAGAA*CACGTG*AATTTTTTATTGATTGC-3'
	5'-GCAATCAATAAAAAATTCACGTGTTCTCCAATGGTGCGACCAAACAACTTCTATTACCAA-3'
BdSAUR50 (Bradi1g51490)	5'-GCATGAATGCACGTACGGAGTTTGGTACGCATG*CATGTG*TACATGTAGTGTACGTGAATG-3'
	5'-CATTCACGTACACTACATGTACACATGCATGCGTACCAAACTCCGTACGTGCATTCATGC-3'

[a]The restriction enzymes used for cloning are underlined.
[b]The G-box (CACGTG; *BdMIR156H*), E-box (CANNTG; *BdSAUR50*), and N-box (CACG(A/C)G; BdPOR) motifs used for the binding assays of BdPIL1/BdPIL3 are underlined and shown in italics.

Supplementary Table S2. Selection of the homozygous lines for the RNAi-suppression of *Bradi1g13980* (*BdPIL1*).[a]

Line No.		Generation	# of survived plants	# of death plant	χ^2	Ratio
#1		**T2**	**8**	**3**	**0.275482**	**3:1**
	1-4	T3	23	0		(Homo)
	1-5	T3	23	0		(Homo)
#7		**T2**	**24**	**7**	**0.312175**	**3:1**
	7-1	T3	28	0		(Homo)
	7-4	T3	34	0		(Homo)
	7-5	T3	32	0		(Homo)
	7-7	T3	24	0		(Homo)
	7-10	T3	28	0		(Homo)
#8		**T2**	**25**	**8**	**0.030609**	**3:1**
	8-2	T3	34	0		(Homo)
	8-3	T3	28	0		(Homo)
	8-5	T3	26	0		(Homo)
	8-6	T3	26	0		(Homo)
	8-9	T3	36	0		(Homo)
#9		**T2**	**21**	**8**	**0.356718**	**3:1**
	9-1	T3	37	0		(Homo)
	9-4	T3	35	0		(Homo)
	9-8	T3	38	0		(Homo)
	9-10	T3	34	0		(Homo)
	9-20	T3	40	0		(Homo)

[a]3:1 segregation ratio was analyzed to obtain the homozygous lines (Homo) used in this study.

Supplementary Table S3. Selection of the homozygous lines for the RNAi-suppression of *Bradi2g11100* (*BdPIL3*).[a]

Line No.		Generation	# of survived plants	# of death plant	χ^2	Ratio
#2		**T2**	**20**	**7**	**0.045725**	**3:1**
	2-1	T3	26	0		(Homo)
	2-4	T3	35	0		(Homo)
	2-6	T3	28	0		(Homo)
	2-8	T3	25	0		(Homo)
	2-10	T3	36	0		(Homo)
#3		**T2**	**17**	**7**	**0.925926**	**3:1**
	3-3	T3	35	0		(Homo)
	3-13	T3	38	0		(Homo)
#4		**T2**	**23**	**7**	**0.148148**	**3:1**
	4-2	T3	34	0		(Homo)
	4-10	T3	28	0		(Homo)
	4-11	T3	26	0		(Homo)
	4-16	T3	26	0		(Homo)
#5		**T2**	**30**	**9**	**0.197239**	**3:1**
	5-2	T3	37	0		(Homo)
	5-5	T3	35	0		(Homo)
	5-16	T3	38	0		(Homo)
	5-17	T3	34	0		(Homo)
#8		**T2**	**22**	**8**	**0.148148**	**3:1**
	8-1	T3	35	0		(Homo)
	8-8	T3	11	0		(Homo)
	8-12	T3	33	0		(Homo)
	8-18	T3	34	0		(Homo)
#11		**T2**	**16**	**5**	**0.075586**	**3:1**
	11-2	T3	45	0		(Homo)
	11-4	T3	48	0		(Homo)
	11-10	T3	43	0		(Homo)
#16		**T2**	**22**	**8**	**0.148148**	**3:1**
	16-5	T3	38	0		(Homo)

[a]3:1 segregation ratio was analyzed to obtain the homozygous lines (Homo) used in this study.

Supplementary Table S4. Genes used in this study.

LOC	ID[1]	Protein identity[2]
LOC100828542	Bradi1g13980	transcription factor PIF1 (BdPIL1)
LOC100844358	Bradi2g11100	transcription factor APG (BdPIL3)
LOC100836209	Bradi1g10520	phytochrome A type 3 (BdphyA)
LOC100829838	Bradi1g64360	phytochrome B (BdphyB)
LOC100838644	Bradi1g28120	auxin-responsive protein SAUR36 (BdSAUR36)
LOC100828255	Bradi1g51490	auxin-responsive protein SAUR50 (BdSAUR50)
LOC100830094	Bradi2g24980	gibberellin 20 oxidase 2 (BdGA20ox2-1)
LOC100823873	Bradi2g57027	gibberellin 20 oxidase 2 (BdGA20ox2-2)
LOC100840545	Bradi3g46930	cell number regulator 2 (BdCNR2)
LOC104584443	Bradi4g10290	cell number regulator 13 (BdCNR13)
LOC100830331	Bradi3g15490	zinc finger protein CONSTANS-LIKE 4 (BdCOL4)
LOC100823662	Bradi1g43220	zinc finger protein CONSTANS-LIKE 9 (BdCOL9)
LOC100842079	Bradi1g08340	MADS-box transcription factor 14-like (BdVRN1; Feng et al., 2017)
LOC100830296	Bradi1g43671	zinc finger protein HD1 (BdCO1; Qin et al., 2019)
LOC100825617	Bradi3g41500	zinc finger protein CONSTANS-LIKE 2 (BdCOL2)
LOC100842660	Bradi3g57000	zinc finger protein CONSTANS-LIKE 16 (BdCOL16)
LOC100824109	Bradi3g19011	zinc finger protein CONSTANS-LIKE 14 (BdCOL14)
LOC100831221	Bradi1g48830	protein HEADING DATE 3A (BdFT1; Lv et al., 2014)
LOC100831513	Bradi1g19220	magnesium-chelatase subunit ChlH (BdChlH)
LOC100836032	Bradi3g05160	porphobilinogen deaminase (BdPBGD)
LOC100841654	Bradi3g30160	glutamyl-tRNA reductase 2 (BdHEMA1)
LOC100826370	Bradi1g49770	magnesium-chelatase subunit ChlI (BdChlI)
LOC100824873	Bradi5g26230	protochlorophyllide reductase (BdPOR)
LOC104794734	MIR156H	BdMIR156H

[1]Gene ID was obtained from *B. distachyon* genome using the BLAST search.
[2]The protein identity was obtained from the NCBI search using the LOC numbers.
